# Toxicological Responses of α-Pinene-Derived Secondary Organic Aerosol and Its Molecular Tracers in Human Lung Cell Lines

**DOI:** 10.1021/acs.chemrestox.0c00409

**Published:** 2021-03-03

**Authors:** Faria Khan, Karina Kwapiszewska, Yue Zhang, Yuzhi Chen, Andrew T. Lambe, Agata Kołodziejczyk, Nasir Jalal, Krzysztof Rudzinski, Alicia Martínez-Romero, Rebecca C. Fry, Jason D. Surratt, Rafal Szmigielski

**Affiliations:** † Institute of Physical Chemistry, Polish Academy of Sciences, 00Kasprzaka 44/52, 01-224 Warsaw, Poland; ‡ Department of Environmental Sciences and Engineering, Gillings School of Global Public Health, University of North Carolina at Chapel Hill, Chapel Hill, North Carolina 27599, United States; § Aerodyne Research Inc, Billerica, Masachusetts 01821, United States; ∥ TROPOS, Leibniz-Institut für Troposphärenforschung, Permoserstrasse 15, 04318 Leipzig, Germany; ⊥ Department of Interdisciplinary Science, Nanjing University of Information Science & Technology, Nanjing, Jiangsu 210044, P. R. China; # Cytomics Core Facility, Príncipe Felipe Research Center, Avenida Eduardo Primo Yúfera, 3, Valenica 46012, Spain; ∇ Department of Chemistry, University of North Carolina at Chapel Hill, Chapel Hill, North Carolina 27599, United States

## Abstract

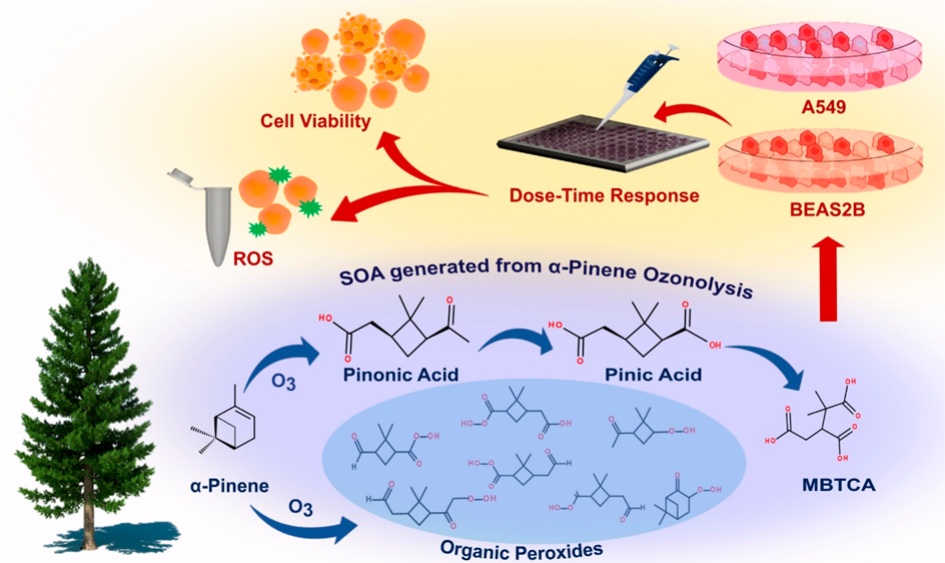

Secondary organic aerosol (SOA) is a major component of airborne fine particulate matter (PM_2.5_) that contributes to adverse human health effects upon inhalation. Atmospheric ozonolysis of α-pinene, an abundantly emitted monoterpene from terrestrial vegetation, leads to significant global SOA formation; however, its impact on pulmonary pathophysiology remains uncertain. In this study, we quantified an increasing concentration response of three well-established α-pinene SOA tracers (pinic, pinonic, and 3-methyl-1,2,3-butanetricarboxylic acids) and a full mixture of α-pinene SOA in A549 (alveolar epithelial carcinoma) and BEAS-2B (bronchial epithelial normal) lung cell lines. The three aforementioned tracers contributed ∼57% of the α-pinene SOA mass under our experimental conditions. Cellular proliferation, cell viability, and oxidative stress were assessed as toxicological end points. The three α-pinene SOA molecular tracers had insignificant responses in both cell types when compared with the α-pinene SOA (up to 200 μg mL^–1^). BEAS-2B cells exposed to 200 μg mL^–1^ of α-pinene SOA decreased cellular proliferation to ∼70% and 44% at 24- and 48-h post exposure, respectively; no changes in A549 cells were observed. The inhibitory concentration-50 (IC_50_) in BEAS-2B cells was found to be 912 and 230 μg mL^–1^ at 24 and 48 h, respectively. An approximate 4-fold increase in cellular oxidative stress was observed in BEAS-2B cells when compared with untreated cells, suggesting that reactive oxygen species (ROS) buildup resulted in the downstream cytotoxicity following 24 h of exposure to α-pinene SOA. Organic hydroperoxides that were identified in the α-pinene SOA samples likely contributed to the ROS and cytotoxicity. This study identifies the potential components of α-pinene SOA that likely modulate the oxidative stress response within lung cells and highlights the need to carry out chronic exposure studies on α-pinene SOA to elucidate its long-term inhalation exposure effects.

## 1 Introduction

Airborne fine particulate matter (PM_2.5_, aerosol particles with aerodynamic diameters ≤2.5 μm) contributes to poor air quality and visibility degradation, in addition to playing a key role in the climate system^1,2^ and in adverse human health effects.^3,4^ PM_2.5_ is linked to human health effects ranging from exacerbation of asthma symptoms to mortatiliy associated with lung cancer and cardiopulmonary disease.^5,6^ In addition, PM_2.5_ has been associated with negative health outcomes with an estimated contribution of more than 103 million indirect disabilities^7^ and 9 million premature deaths in 2015 worldwide.^8,9^ Even though there is some evidence that PM_2.5_ composition affects toxicity in cell lines of lung origin, fewer studies focus on the link between PM_2.5_ chemical composition and biological outcomes associated with its exposures.^10^

Secondary organic aerosol (SOA) is one of the largest mass fractions of PM_2.5_ and is formed from the atmospheric oxidation of volatile organic compounds (VOCs) by ozone (O_3_), hydroxyl radical (^•^OH), and nitrate radicals (NO_3_^•^).^1^ Emissions of both biogenic (derived from terrestrial vegetation) and anthropogenic VOCs contribute to SOA formation through the nucleation, condensation, or multiphase reactions of their semi- and/or low-volatility atmospheric oxidation products.^1,2,11^ Monoterpene (C_10_H_16_) emissions contribute up to 15% of the total biogenic VOCs emitted into the troposphere each year,^12,13^ with α-pinene being the most abundant monoterpene from tree emissions.^14,15^ The global emission rate of α-pinene varies with the vegetation type and geographical location; however, its average emission is estimated to be 66 Tg yr^–1^.^16^ Because of its high emission rate and SOA yield,^1,11^ prior studies have begun examining how exactly α-pinene-derived SOA may adveresly affect human health;^17−25^ However, detailed toxicological properties of α-pinene SOA and/or individual molecular tracers associated with this SOA type are not currently available.

First-generation oxidation products of α-pinene that have been measured in SOA include pinonaldehyde as well as pinic, pinonic, and 10-hydroxypinonic acids.^26−28^ Another important molecular marker for α-pinene-derived SOA formation is 3-methyl-1,2,3-butanetricarboxylic acid (MBTCA), which is formed via ^•^OH oxidation of pinonic acid.^29,30^ In addition to aldehydes and carboxylic acids, α-pinene SOA may contain organic peroxides,^31^ dimer esters,^32,33^ organosulfates,^34^ and/or extremely low-volatility organic compounds (ELVOCs).^15^ ELVOCs are believed to form from the autoxidation of first-generation peroxy radicals (RO_2_^•^) generated by either α-pinene + O_3_ or α-pinene + ^•^OH reactions.^15^ These ELVOCs may generate toxicological effects within human lung cells upon inhalation to α-pinene SOA,^21^ especially since prior studies have demonstrated that multifunctional organic hydroperoxides form within SOA.^15^ Owing to the low-volatility, monoterpene SOA constituents within PM_2.5_^35,36^ may have atmospheric lifetimes of ∼2 weeks,^2^ and consequently can result in inhalation exposures by populations living in close proximity or downwind of their initial formations.

Recent studies have demonstrated that pro-inflammatory and inflammatory-related genes can be activated within lung cells when exposed to PM_2.5_.^37^ Chemical-based assays, such as dithiothreitol (DTT) consumption, have measured the oxidative stress of biogenic SOA.^38,39^ In vitro and in vivo studies have measured the expression of pro-inflammatory protein biomarkers, such as interleukin-8 (IL-8), interluekin-6 (IL-6), and tumor necrosis factor alpha (TNF-α), upon exposure to α-pinene SOA.^22−24^ Evidence of oxidative stress build-up illustrates the underlying cellular pathophysiology contributing to chronic and acute lung diseases.^40,41^ Repeated or prolonged exposures to the pollutant particles can trigger obstructive lung diseases, resulting in increased morbidity and overall decreased quality of life.^42^ A prior study reported the use of a deposition chamber to expose α-pinene SOA to lung macrophages for up to 2 h, which did not induce observable cytotoxicity.^20^ Another study demonstrated that the ROS content of α-pinene SOA increases with its corresponding photochemical age,^18^ suggesting a potential association between SOA chemical composition and toxicity that is not currently understood. Increasing concentration response and subsequent changes in the biological pathway can help us determine the health effects of certain inhalable aerosol particles.^7^ This is in part because the concentration and time-dependent response following exposure to individual molecular components of α-pinene SOA and comparison with the full mixture of SOA generated from α-pinene ozonolysis has not been reported in the literature.

In recent years, changes in precursor molecule emissions from anthropogenic sources has increased PM_2.5_ emission/formation and inadvertantly contributed toward climatic change.^43^ The global climatic change has also resulted in higher emission rates of biogenic VOCs due to increased temperatures and changes in plant metabolism.^44,45^ The resultant increase in atmospheric α-pinene SOA concentration require development of standards on minimal safe inhalation concentration (MSIC) from a public health perspective.^17^ The objective of the present study was to determine the concentration and time-dependent responses of lung cells to known α-pinene SOA molecular tracers (i.e., pinic acid, pinonic acid, and MBTCA) and to the full SOA mixture generated from α-pinene ozonolysis. To understand post-exposure responses, an immortalized normal bronchial epithelial (BEAS-2B) cell line and a cancer alveolar epithelial (A549) cell line were used as the lung cell models in the current study.^46^ This comparison allowed us to investigate the potential toxicity of α-pinene-derived SOA compounds, including for multifunctional organic peroxides that could not be examined individually due to the lack of available authentic standards but could be examined as a mixture within the full SOA generated from α-pinene ozonolysis.

## 2 Materials and Methods

### 2.1 Chemicals

MBTCA (3-methyl-1,2,3-butanetricarboxylic acid, 99% purity) and *cis*-pinic acid (99.5% purity) were synthesized in our laboratory, as described previously.^47,48^ α-Pinene (>99% purity), *cis*-pinonic acid (98% purity), dimethyl sulfoxide (DMSO, MolBio-Grade), Dulbecco’s phosphate-buffered saline (PBS), methanol (ChromaSolv-Grade), and 0.1% acetic acid were purchased from Sigma-Aldrich (Merck, Poland). High-purity methanol (optima LC/MS grade) was purchased from Fisher Chemical (U.S.A.). A Milli-Q water advantage system (Merck, Poland) was used to obtain Milli-Q water (resistivity 18.2 MΩ × cm at 25 °C) to dissolve all the standards and probes.

### 2.2 Fluorescent Probes and Assays

All assays and fluorescent probes, including the MTT assay kit (3-(4,5-dimethylthiazol-2-yl)-2,5-diphenyltetrazolium bromide), calcein-AM (a live cell marker), propidium iodide (PI, a fluorescent DNA intercalating probe), and carboxy-dihydrodichlorofluoresceine diacetate (carboxy-H_2_DCFDA, a general oxidative stress indicator), were purchased from Invitrogen (ThermoFisher Scientific, U.S.A.). Trypan blue solution and Triton X-100 solutions were purchased from Sigma-Aldrich (Poland).

### 2.3 Cell Culture and Medium

BEAS-2B (ATCC CRL-9609) and A549 (ATCCCCL-185) cell lines were purchased from ATCC. For culturing BEAS-2B cells, ATCC recommended the bronchial epithelial cell growth medium (BEGM), complete with supplements and growth factors (BEpiCM, ScienCell, U.S.A.).

Depending on the experiment, A549 cells were cultured in Dulbecco’s modified eagle’s medium (DMEM, Institute of Immunology and Experimental Technology, Wrocław, Poland) or phenol-red free DMEM in 5% or 10% heat-inactivated fetal bovine serum (FBS), l-glutamine (L/G, 2 mM) and penicillin-streptomycin (P/S, 100 mg mL^−1^). All chemicals were purchased from Sigma-Aldrich (U.S.A.). Trypsin-EDTA (0.25% solution with phenol red, Sigma-Aldrich, U.S.A.) was used to routinely detach the adherent cells for passaging. Both cell lines were cultured in tissue culture (TC) treated T75 flasks (Cell Star, Greiner Bio-One, Austria) and maintained at 37 °C in a 5% CO_2_ humidified incubator during the course of the experiment. Depending on the type of the experiment, cells were seeded onto 96-well advanced TC treated μclear microplates (Greiner Bio-One, Austria). For all experiments, BEAS-2B cells underwent 1–16 passages, while A549 cells underwent 3–20 passages.

### 2.4 Generation of α-Pinene SOA

SOA from α-pinene ozonolysis was produced in a Potential Aerosol Mass (PAM) oxidation flow reactor (Aerodyne Research, Inc.)^49−51^ under dry (relative humidity, RH < 5%) and dark conditions. A syringe pump (Chemyx, Fusion 100) was employed to continuously inject α-pinene mixed with 3 L min^–1^ clean dry N_2_ (g) inside a glass bulb to entrain the vapor into the reactor. At the injection rate that was used, the α-pinene mixing ratio was approximately 5 ppm. A separate flow of 2 L min^–1^ filtered clean air was introduced into a separate O_3_ generator to produce 30 ppm of O_3_ that was subsequently injected to the reactor. Following SOA generation in the reactor, the sample flow was passed through activated charcoal denuders and an O_3_ denuder to remove excess α-pinene and O_3_, respectively. SOA number concentrations and mobility size distributions were obtained with a scanning mobility particle sizer (SMPS, TSI Inc., Model 3080). Integrated filter samples were obtained following collection of SOA particles onto prebaked 47 mm quartz-fiber filters at 3 L min^–1^ (Tissuquartz, Pall Life Sciences, treated at 600 °C for 24 h). Typical SOA mass concentrations were approximately 6 mg m^–3^, which was estimated from SMPS measurements assuming a particle density of 1.2 g cm^–3^.^52^ At these concentrations, 20 mg filter samples were obtained following 18.5 h of continuous collection. While significantly lower SOA concentrations are easily achieved in the reactor, the associated longer collection times necessary to achieve 20 mg samples were not practical. After collection, filters were stored under dark conditions at −20 °C until analysis.

### 2.5 RPLC/ESI-HR-QTOFMS Analysis of α-Pinene SOA

The chemical characterization of α-pinene SOA was performed by ultraperformance liquid chromatography interfaced to electrospray ionization high-resolution quadrupole time-of-flight mass spectrometry (UPLC/ESI-HR-QTOFMS, 6520 Series, Agilent) operated in the negative and positive ion modes in order to measure organic acids and organic peroxides, respectively.^53,54^ The α-pinene SOA constituents were extracted from quartz-fiber filters with 10 mL of high-purity methanol by sonication for 45 min. Prior to drying, extracts were filtered through 0.2-μm PTFE syringe filters (Pall Life Sciences, Acrodisc) to remove insoluble particles or quartz filter fibers. The methanol extracts were blown dry under a gentle N_2_ (g) stream at ambient temperature. The dried extracts were reconstituted with 300 μL of a 50:50 (v/v) solvent mixture of methanol and water. The reconstituted extracts were further diluted by a factor of 20 for the purpose of quantifications of three α-pinene SOA tracers (i.e., pinonic acid, pininc acid, and MBTCA). In the negative ion mode, a six-point calibration of the three α-pinene SOA tracers was construed in the range between 0.5 and 50 ppm (Supporting Information, SI, Figure 7) and was analyzed along with the SOA samples produced from α-pinene ozonolysis. It is noted here from the 6-point calibration that the three α-pinene SOA tracers showed the linear response only up to 10 ppm, and that the samples fell within this linear dynamic range. 5-μL aliquots of the calibration standards and samples were injected onto the UPLC column (Waters ACQUITY UPLC HSS T3 column, 2.1 × 100 mm^2^, 1.8-μm particle size) at a flow rate of 0.3 mL min^–1^; the UPLC column was a reversed-phase liquid chromatography (RPLC) column, and thus, we will refer to this method as RPLC/ESI-HR-QTOFMS hereafter. The mobile phases consisted of (A) 0.1% acetic acid in Milli-Q water, and eluent (B) 0.1% acetic acid in methanol (Optima LC/MS grade). The applied 15 min gradient elution program was as follows: the concentration of eluent B was 0% for the first 2 min, increased to 90% from 2 to 10 min, held at 90% from 10 to 11 min, and decreased to 0% from 11 to 15 min. Detailed operating procedures (e.g., mass calibration, tuning, voltages, etc.) of the RPLC/ESI-HR-QTOFMS method have been previously published.^44−47^ The same mobile phases and gradient elution program were employed for RPLC/ESI-HR-QTOFMS analysis operated in the positive ion mode in order to aid in the detection of additional α-pinene SOA constituents, including organic peroxides (hydroxyhydroperoxides). The injection volume used for positive ion mode was 10 μL. Previously, we have demonstrated that isoprene-derived hydroxyhydroperoxides (1-2-ISOPOOH; specifically, 2-hydroperoxy-2-methylbut-3en-1-ol) synthesized at UNC could be measured by the RPLC/ESI-HR-QTOFMS method operated in the positive ion mode.^55^ As a result, we injected the 1,2-ISPOOH standard synthesized at UNC onto the RPLC/ESI-HR-QTOFMS to demonstrate how organic hydroperoxides breakdown during tandem mass spectrometric (MS/MS) experiments in the positive ion mode (SI Figure 8). This aided in the structural characterization of potential organic hydroperoxides measured in the present study from SOA generated from α-pinene ozonolysis. Similarly, in the positive ion mode, a six-point calibration of the 1,2-ISOPOOH was construed in the range between 0.5 and 50 ppm (SI Figure 8(d)) and was analyzed along with the SOA samples produced from α-pinene ozonolysis.

### 2.6 α-Pinene SOA Filter Extraction for Toxicological Studies

Quartz-fiber filters were sonicated twice each for 30 min in 22 mL of high-grade methanol in a precleaned 22 mL scintilation vial. The methanol extracts were blown dry under a gentle N_2_ (g), as followed in Section 2.5; ∼2 mg of mass was reconstituted in 1 mL of deionized water prior to the exposure studies with the two cell types.

### 2.7 MTT Assay for Cellular Proliferation

Cells were seeded at 5000–8000 cells well^–1^ for A549, and 10 000–12 000 cell well^–1^ for BEAS-2B; these were allowed to adhere for at least 16 h in 96-well plates. Cells were counted using trypan blue in automated Countess II Automated Cell Counter (Thermo Fisher Scientific). The cells were then replenished with 100 μL of fresh medium (complete BEGM for BEAS-2B cells, or DMEM (phenol-red free, reduced serum medium) supplemented with 5% FBS, 1% P/I, 1% L/G for A549 cells), and treated with various doses of 10 μL of individual α-pinene SOA molecular tracers (i.e., pinonic acid, pinic acid and MBTCA), a 3-component mixture of the same α-pinene SOA molecular tracers that had authentic standards (equal concentration/volume ratio mixture), or the α-pinene ozonolysis SOA sample (all dissolved and prepared in double distilled and deionized water). Following 24 and 48 h of exposure time, the medium was decanted from all the wells and fed with 100 μL of fresh medium, followed by the addition of 10 μL of 5 mg mL^–1^ of MTT dye (dissolved in 1× PBS). Cells were incubated at 37 °C from 2 to 3 h until they formed NAD(P)H-oxidoreductase reduced formazan insoluble crystals. The crystals were dissolved in 100 μL well^–1^ of DMSO as a solubilizing agent and allowed to incubate for another 10 min. The end-point absorbance was recorded using spectrophotometer (BioTex Synergy HTX) at 540 nm with the background signal (DMSO) subtracted from the recorded readings. Final calculations for cellular proliferation rate were made as follows:
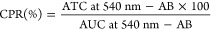
where CPR is the cellular proliferation rate, ATC is the absorbance of treated cells, AUC is the absorbance of untreated cells, and AB is the absorbance of the blank, with untreated cells as the control without any aerosol treatment while the blank is the medium without any cells.

### 2.8 Live–Dead Cell Stain Imaging

The live–dead cell stain imaging was performed using calcein AM (1 mg mL^–1^ dissolved in DMSO for live cells) and propidium iodide (PI, 1 mg mL^–1^ in DI water for dead cells). The final calcein AM:PI ratio of the fluorescent probe dissolved in PBS was 1:10. The fluorescent probe was added directly to 24- and 48-h treated cells, respectively in 96 well plates without media removal. Calcein-AM is a cell permeant nonfluorescent dye that converts into a fluorescent form through acetoxymethyl ester hydrolysis by cellular sterases. This sterase activity is a marker of cell vaibility, rendering a green fluorescent and charged form of the probe that is well-retained in live cells. PI is a nonpermeant DNA marker, unable to stain live cells, and only the cells with the damaged cell membrane (dead cells) appear as positive for PI. Images were acquired using fluorescence microscopy (Nikon Eclipse T1-SAM, Japan) in the TRITC and FITC filter range (Ti-FLC filters) at ×100 magnification. High-resolution images were captured using Nikon DS-U2 digital sight (Japan). While capturing the image, cells were scanned at 6.8 gain settings, resolution of captured images fixed at fast-focus mode of 640 × 480 pixels. The scan exposure in the calcein-AM channel was kept fixed at 200 ms, while cells in PI channnel were scanned at exposure of 400 ms. During the course of an experiment, the plates were kept on the stage and maintained at 37 °C using Linkam DC-60 thermo-controller. NIS-Elements imaging software was used for initial imaging, while the final images were analyzed with NCBI’s Image-J software, a freely available software at https://imagej.net/Fiji. All the images were captured at a fixed area of 600 × 800 μm^2^ using 10× objective lens in three modes: calcein-AM + filter channel, PI+ filter channel and phase-contrast brightfield mode. The PI+ BEAS-2B cells were calculated using particle size analysis function of image-J. The α-pinene SOA treated cells were normalized to untreated control cells population by capturing cellular density in the fixed area (4.8 × 10^4^ μm^2^), as determined in three independent experiments.

### 2.9 Oxidative Stress Studies using the Carboxy-H2DCFDA Assay

For the flow cytometry analysis, 20 000 cells well^–1^ for BEAS-2B and 10 000 cell well^–1^ for A549 were seeded. Cells were analyzed 6 h postaerosol exposure. Conditioned cell medium was collected in prelabeled centrifuge tubes, and the wells were washed once with 50 μL of 1× PBS and collected again in centrifuge tubes. The cells were then trypsinized for 5–10 min using 50 μL of Trypsin-EDTA and collected in centrifuge tubes as well. Cell suspension was then centrifuged at 300*g* (gravity) for 5 min at 25 °C. Supernatant was discarded, cells resuspended in 100 μL of fresh medium plus 100 uL of 10 μM of carboxy-H_2_DCFDA and 1 μg mL^–1^ PI in 1× PBS. Treated (experimental) and untreated (control) cell groups (BEAS-2B and A549) were analyzed for an increased oxidative stress signal using a CytoFLEX S Flow Cytometer (Beckman Coulter, U.S.A.) and data recorded using CytExpert software.

Carboxy-H_2_DCFDA is a nonfluorescent cell permeant probe. Upon deacetylation by cellular sterases and oxidation by ROS, the molecule is converted into carboxy-DCF, which has green fluorescence emission. The carboxy-DCF signal was analyzed in live and single cells using laser 488 nm excitation and the 525 nm emission of the CytoFLEX S. First, the agreggates were discarded using the forward scatter area (FSA) and height (FSH) signals, defining a “single cells” gate. PI signal was detected using the laser 561 nm excitation and 610 nm emission of the CytoFLEX S. PI+ cells were defined as dead cells and were discarded from the analysis, defining a “live cells” gate. The double gate “live and single cells” was applied to the carboxy-DCF measurement.

### 2.10 Statisitical Analyses

Data were analyzed for normality assumptions using one-way Anova and are presented as mean ± standard deviation (SD) of at least three independent repetitions. For the concentration time-dependent MTT data, results were analyzed by using a one-way ANOVA followed by Tukey’s posthoc test; these statistical analyses were performed using GraphPad Prism (Version 8.00 for Windows, GraphPad Software, La Jolla California U.S.A., www.graphpad.com). The results were considered statistically significant at *p*-value ≤0.05 for all exposure groups at two time points. The Tukey’s test allowed us to analyze the difference in the mean of treatment groups with the mean of untreated control cells. The significant difference at a particular concentration is reported here as ^+^24 h significant, ^++^48 h significant, and ^+++^significant difference among treatments at 24 and 48 h.

For image-J analyzed PI+ cells in α-pinene SOA treated SOA, the two way Anova with Šídák’s multiple comparisons test was performed on the data set to determine the statistical significance between treated and untreated control cells, where the *p* ≤ 0.05 value was considered significant.

For the in vitro flow cytometry data, the exposures were repeated three separate times (*n* = 3) and were analyzed by repeated measures of one-way ANOVA followed by Dunnett’s posthoc test. A significant difference was determined between the mean of treatment groups with the untreated control cells and reported as ***p ≤ 0.001.

## 3 Results

### 3.1 Time-Dependent Increasing Concentration Response (ICR) Using the MTT Assay

**Figure 1:**
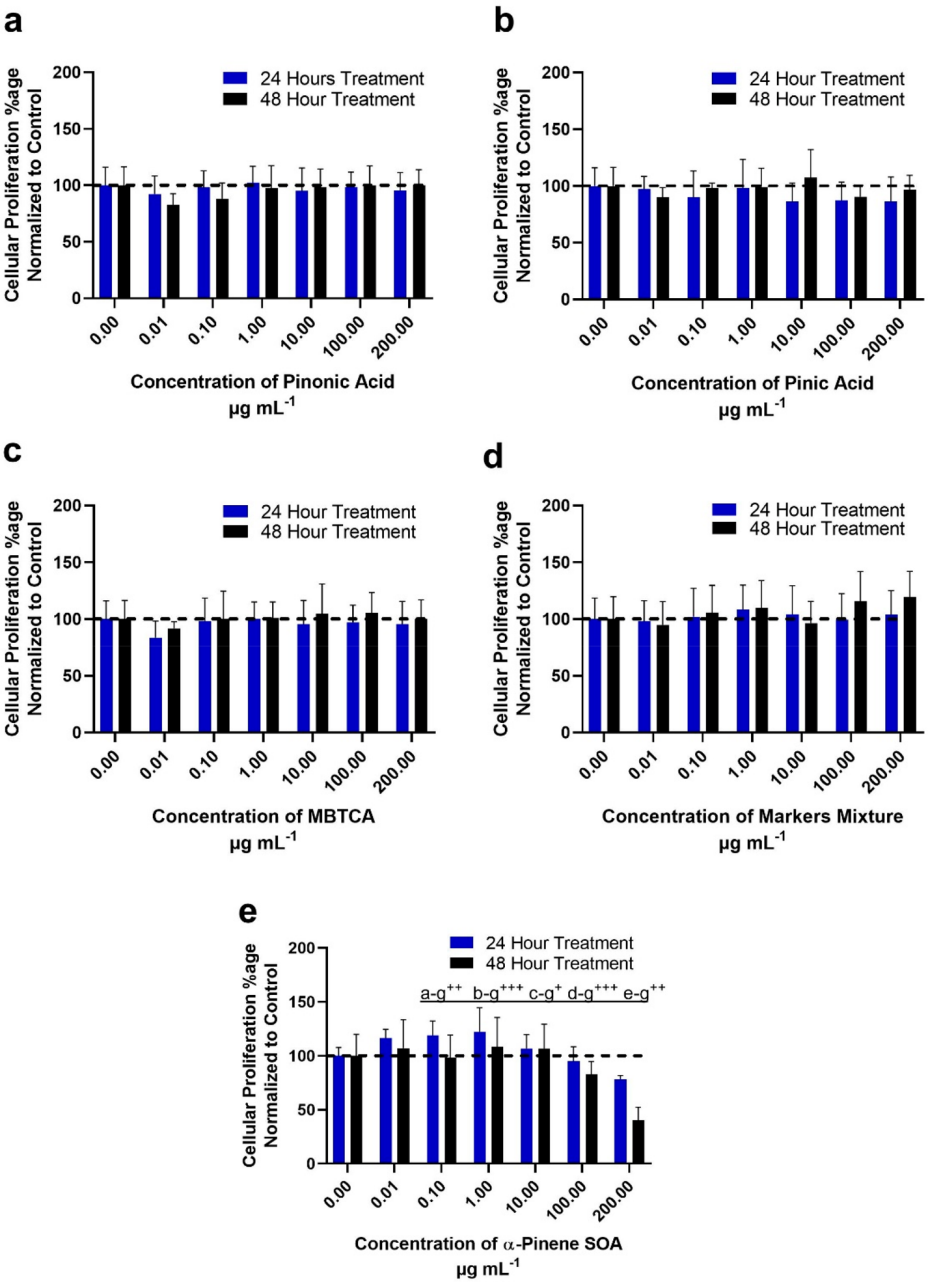
Percentage of cellular proliferation for BEAS-2B cells following treatment with pinonic acid/pinic acid/MBTCA and α-pinene SOA as determined through the MTT assay. The graphs show time-dependent concentration response at 24 and 48 h post exposure of: (a) pinonic acid; (b) pinic acid; (c) MBTCA; (d) equimolar mixtures of pinonic and pinic acids with MBTCA; and (e) α-pinene ozonolysis SOA. The data is representative of three independnent experiments, normalized to untreated control cells for plotting these graphs. (e) The effect of α-pinene ozonolysis SOA on cellular proliferation; a-g: 0–200 μg mL^–1^, b-g: 0.01–200 μg mL^–1^, c-g: 0.1–200 μg mL, d-g: 1–200 μg mL^–1^, and e-g: 10–200 μg m^–1^ depicts significant difference in mean treatments. *P* ≤ 0.05 where ^+^24 h significant, ^++^ 48 h significant, and ^+++^ significant difference among treatments at 24 and 48 h.

**Figure 2:**
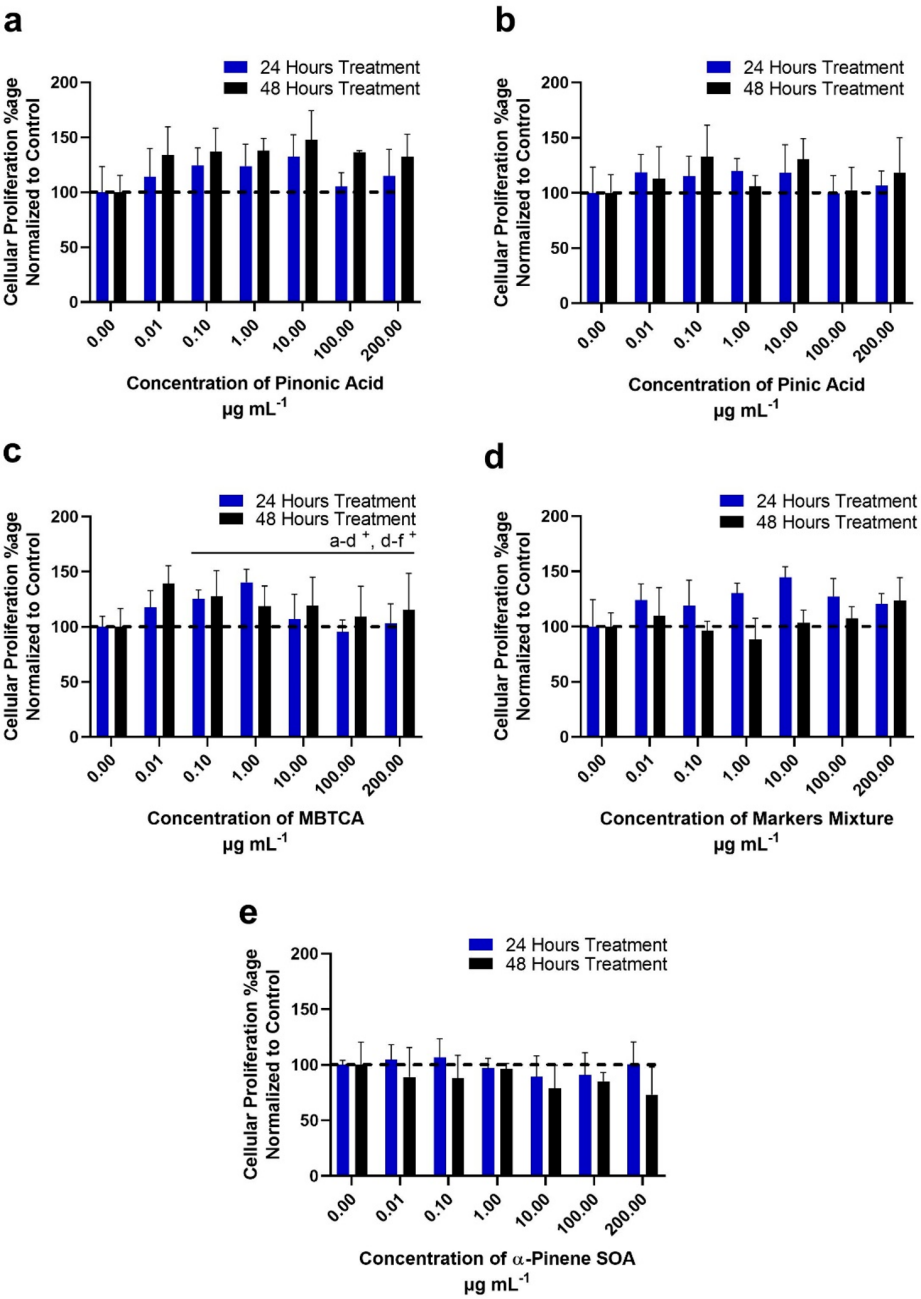
Percentage of cellular proliferation for A549 cells when treated with pinonic acid/pinic acid/MBTCA and α-pinene ozonolysis SOA as determined through the MTT assay. The graphs show time-dependent concentration responses at 24 and 48 h post exposure of the following: (a) pinonic acid; (b) pinic acid; (c) MBTCA; (d) equimolar mixtures of pinonic and pinic acids with MBTCA; and (e) α-pinene ozonolysis SOA. The data are representative of three independnent experiments, which were normalized to untreated control cells for plotting these graphs. Note that A549 cell lines generally exhibited a higher cellular proliferation when compared to BEAS-2B cell lines for each molecular tracer, their mixture, or its full SOA mixture from α-pinene ozonlysis. *P* ≤ 0.05 where ^+^ 24 h treatment significant, and a-d: 0–1 μg mL^–1^ and d-f: 1–100 μg mL^–1^ treatment group difference.

BEAS-2B and A549 cell lines were exposed to three α-pinene SOA molecular tracers (i.e., pinonic acid, pinic acid, and MBTCA), their equimolar mixture (Figures 1d, 2d, S2, and S3), and α-pinene ozonolysis SOA. Exposure concentrations ranged from 0.01 to 200 μg mL^–1^, with changes in cellular proliferation observed at 24- and 48-h post-exposure time points (shown in blue and black bars, respectively, in Figures 1 and 2).

Figure 1 shows the percent change in cellular proliferation normalized to the control in BEAS-2B cells following exposure to the α-pinene SOA molecular tracers, their equimolar mixture, and α-pinene ozonolysis SOA. Notably, the individual α-pinene SOA molecular tracers of pinonic acid (Figure 1a), pinic acid (Figure 1b), and MBTCA (Figure 1c) as well as their equimolar mixture (Figure 1d) did not induce any significantly quantifiable change in the cellular proliferation percentage up to the maximum exposure concentration of 200 μg mL^–1^. However, when BEAS-2B cells were exposed to 200 μg mL^–1^ of the SOA mixture generated from α-pinene ozonolysis (as seen in Figure 1e), the proliferation decreased to 78% (compared to baseline control) after 24 h of exposure and to 44% of untreated control cells after 48 h of exposure. There was no significant difference observed between the proliferation rates observed after 48 h with 100 (82 ± 12%) and 200 μg mL^–1^ (44 ± 12%) exposure concentrations. The calculated inhibitory concentration-50 (IC-_50_) values were 912 and 230 μg mL^–1^ at 24 and 48 h, respectively, in the BEAS-2B cells (SI Figure S1), suggesting increased time of exposure decreased cellular viability.

A549 cells responded differently than BEAS-2B cells when exposed to α-pinene SOA constituents under similar exposure concentration conditions. Unlike BEAS-2B cells, A549 cells did not exhibit statistically significant differences in their proliferation relative to the untreated control following exposure to pinonic acid (Figure 2a), pinic acid (Figure 2b), their equimolar mixture (Figure 2d), and α-pinene ozonolysis SOA (Figure 2e). The only significant change in cellular proliferation percentage (relative to baseline control) was observed when MBTCA was exposed to A549 cells at 24 h. The cellular proliferation rate (metabolism) increased from 100% (untreated control) to 118% after exposure to 0.01 μg mL^–1^ of MBTCA, and then increased further to 124% after exposure to 0.1 μg mL^–1^ and up to 140% after exposure to 1 μg mL^–1^ of MBTCA; the proliferation decreased back to 103% at the 200 μg mL^–1^ exposure concentration of MBTCA. Increased cellular proliferation at the 1 μg mL^–1^ exposure concentration to 140% suggests that cells exhibited metabolic increase with MBTCA in a concentration-dependent manner. Following 48 h exposure to 0.01 μg mL^–1^ of MBTCA, 139% cellular proliferation was observed, indicating a concentration-dependent exposure effect when compared with untreated control cells. Figures S4–S6 (SI) show the phase contrast microscopy images of A549 cells treated with pinonic acid, pinic acid, and MBTCA, respectively, in increasing concentrations (0.01–200 μg mL^–1^). The captured images at 24 and 48 h did not exhibit much increase in cellular population when compared with the untreated control cells. Hence, the increase in MTT activity rate, which is in fact a measure of mitochondrial acticity, as depicted in Figure 2c, did not attribute to increase in cell density following exposure to MBTCA, but rather indicates some mitochondrial metabolic changes within cells, following exposure at low concentrations only.

### 3.3 Live/Dead Cell Staining Assay

**Figure 3:**
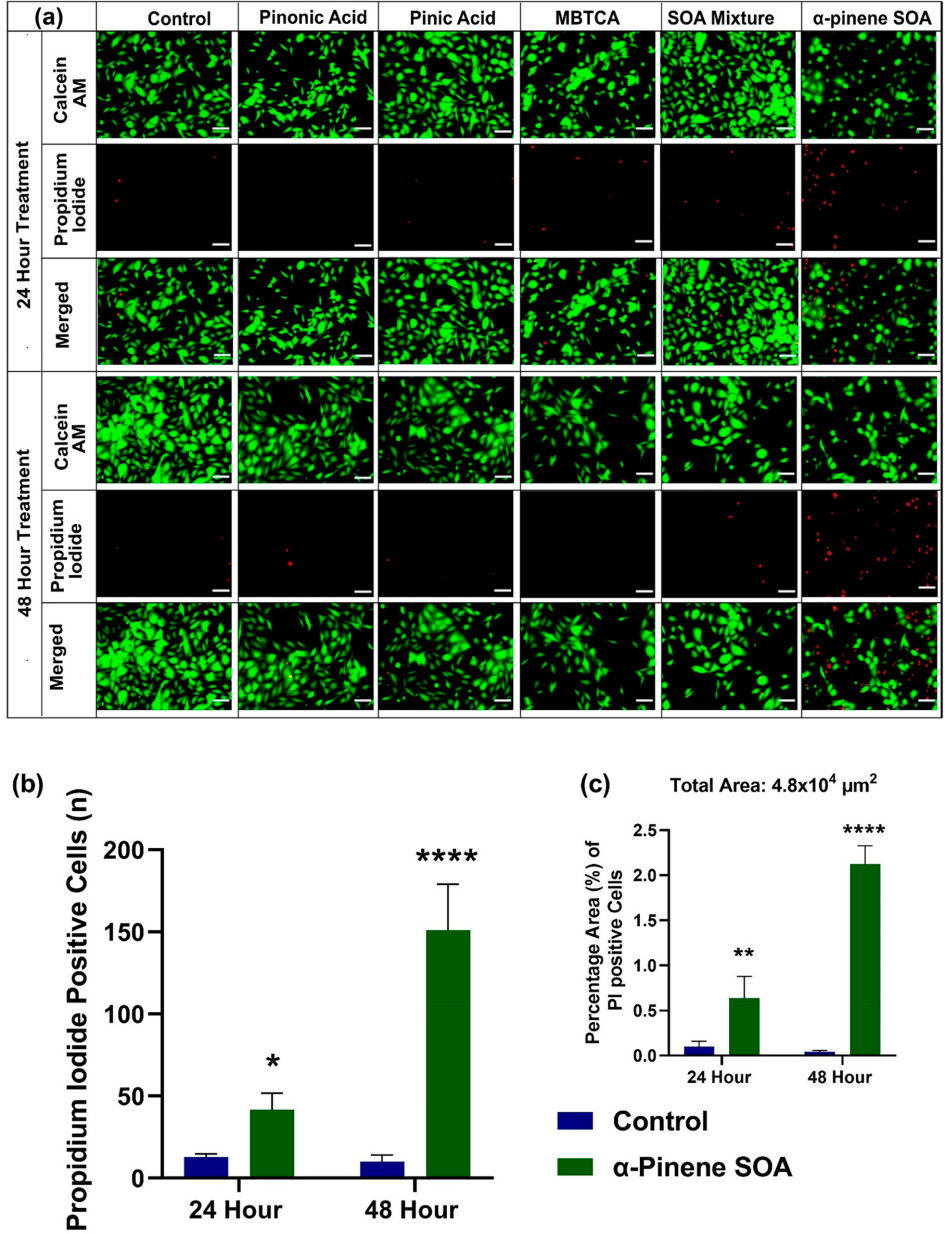
(a) Fluorescent microscopy images of BEAS-2B cells treated with well-established α-pinene SOA molecular tracers (i.e., pinonic acid, pinic acid, and MBTCA), their equimolar mixtures, and the α-pinene ozonolysis SOA at the 200 μg mL^–1^ exposure concentration at 24 h (upper pannels) and 48 h (bottom pannels). The cells were stained with calcein AM (green) for live cells and propidium iodide (red) for dead cells. All the images were scaled to 50 μm, and imaged at ×100 magnification. (b) The graph shows an increase in the number of PI + cell population following treatment with α-pinene SOA when compared with untreated control at 24 and 48 h post exposure, as calculated through the average of three micrographs. (c) The increase in the area percentage (%) of PI + cells in α-pinene SOA compared with untreated cell control. Note that for each experimental and control condition, a fixed area of 600 × 800 μm^2^ was captured for each micrograph shown here. The average of PI+ cells was determined in 4.8 × 10^4^ μm^2^ area, normalized to the same area of the untreated control channel. Two-way Anova with Šídák’s multiple comparisons test was performed on the data set to determine the statistical significance of change in signal when compared with untreated controls. The *p*-value <0.05 was considered statistically significant for our analysis where **** indicates a *p*-value ≤0.0001.

In order to determine whether the changes in cellular proliferation are attributable to decreases in cell number (cell death), or conversely to proliferation inhibition (viability), the calcein-AM/propidium iodide-based staining method was used, and the cells were observed under fluorescent microscopy at ×100 magnification. Figure 3 shows the images of BEAS-2B cells treated with the well-established α-pinene SOA molecular tracers, their equimolar mixtures, and the α-pinene ozonolysis SOA. Notably, the BEAS-2B cells did not undergo any morphological changes when treated with 200 μg mL^–1^ of pinonic acid, pinic acid, MBTCA, and their equimolar mixtures at the 24- and 48-h post-exposure conditions. This was confirmed through little or no staining observed in the propidium iodide (PI) channel, which only stains the nucleus of cells with damaged cell membranes. However, there was increased PI staining when 200 μg mL^–1^ of the α-pinene ozonolysis SOA was added to the proliferating cells (Figure 3b). As exposure time increased from 24 to 48 h, more cells were stained with PI and the BEAS-2B cells appeared more rounded and detached (as observed in the calcein channel). The PI+ micrographs were analyzed through image-J software to calculate that the PI+ cell population increased from 42 ± 10 cells at 24-h treatment to 151 ± 28 cells at 48-h treatment in the total captured area of 4.8 × 10^4^ μm^2^. The untreated control cells had 10 ± 2 and 12 ± 4 PI+ cells at 24 and 48 h, respectively, within the same cellular density area (Figure 3c). This implies that the decrease in cellular proliferation observed in Figure 1e was due to increased cellular death. Hence, both the aerosol exposure concentration and time had a significant role in defining the cellular response in BEAS-2B cells.

**Figure 4:**
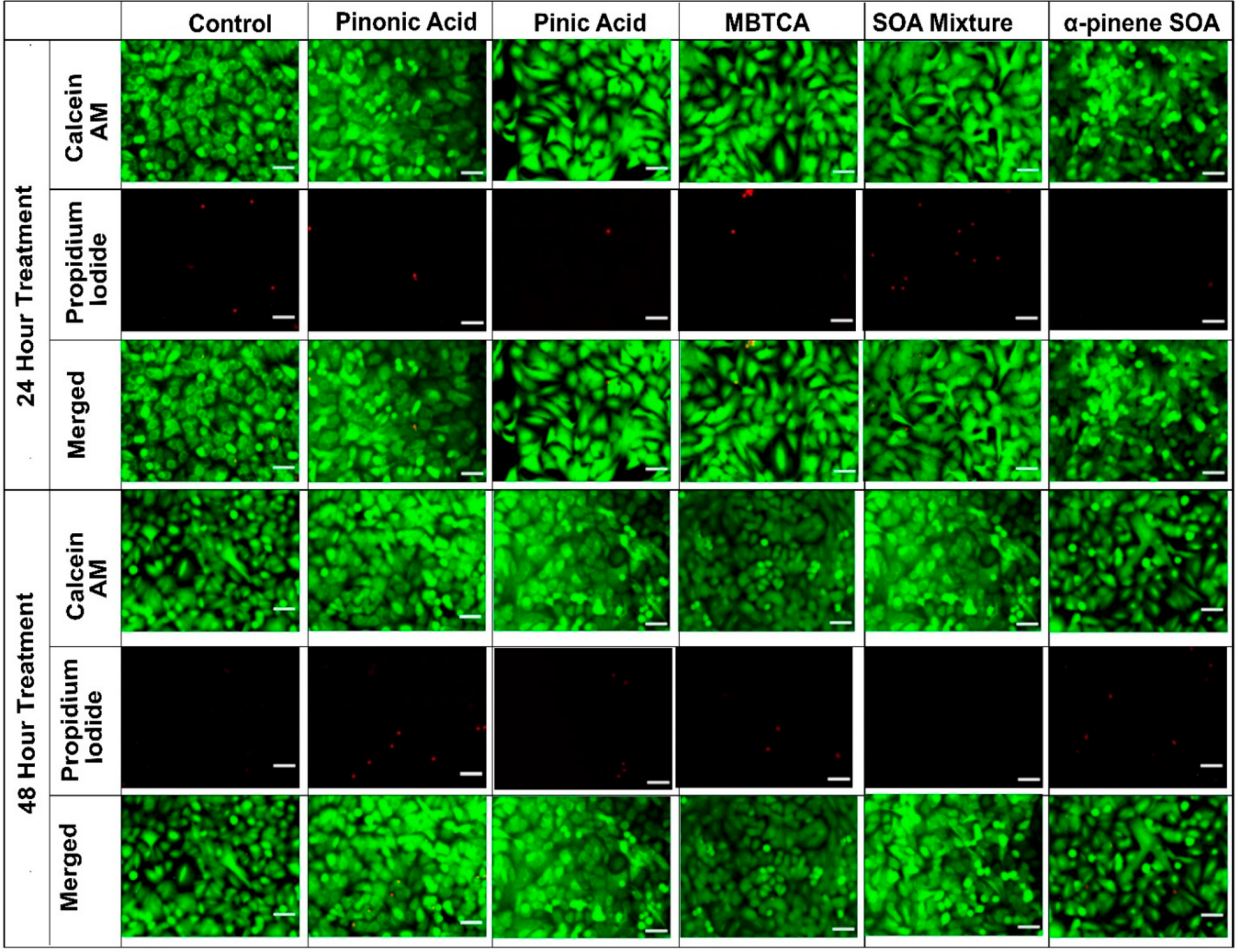
Fluorescent microscopy images of A549 cells treated with pinonic acid/pinic acid/MBTCA and α-pinene SOA at exposure to 200 μg mL^–1^ concentrations for 24 and 48 h. The cells were stained with calcein AM (green) for live cells and propidium iodide (red) for dead cells. Note that there is little to no cell death observed after treatment in A549 cells, and cellular density is not adversely affected even after 48 h of treatment. This implies in cancer lines, the α-pinene SOA causes limited morphological changes. All the images are scaled to 50 μm size, and imaged at ×100 magnification.

However, application of the calcein AM/PI staining method to A549 cells treated with the well-established α-pinene SOA tracers (i.e., pinonic acid, pinic acid, and MBTCA), their equimolar mixtures, and α-pinene ozonolysis SOA, had no significant changes observed in cellular viability. As shown in Figure 4, there was negligible PI staining at the 24 and 48 h post-exposure times. The cells appeared confluent in calcein-AM channel and the number of A549 cells in the treatment groups appeared the same as the untreated controls. This observation is consistent with our MTT assay data in Figure 2, suggesting no apparent change in A549 cell numbers after aerosol exposure. The phase contrast microscopy images of α-pinene ozonolysis SOA treated A549 cells (Figure S7) confirmed the intact morphology, while cells treated with 0.1% Triton-X 100 had damaged cellular membrane.

### 3.4 Oxidative Stress Measurements Using Flow Cytometry

**Figure 5:**
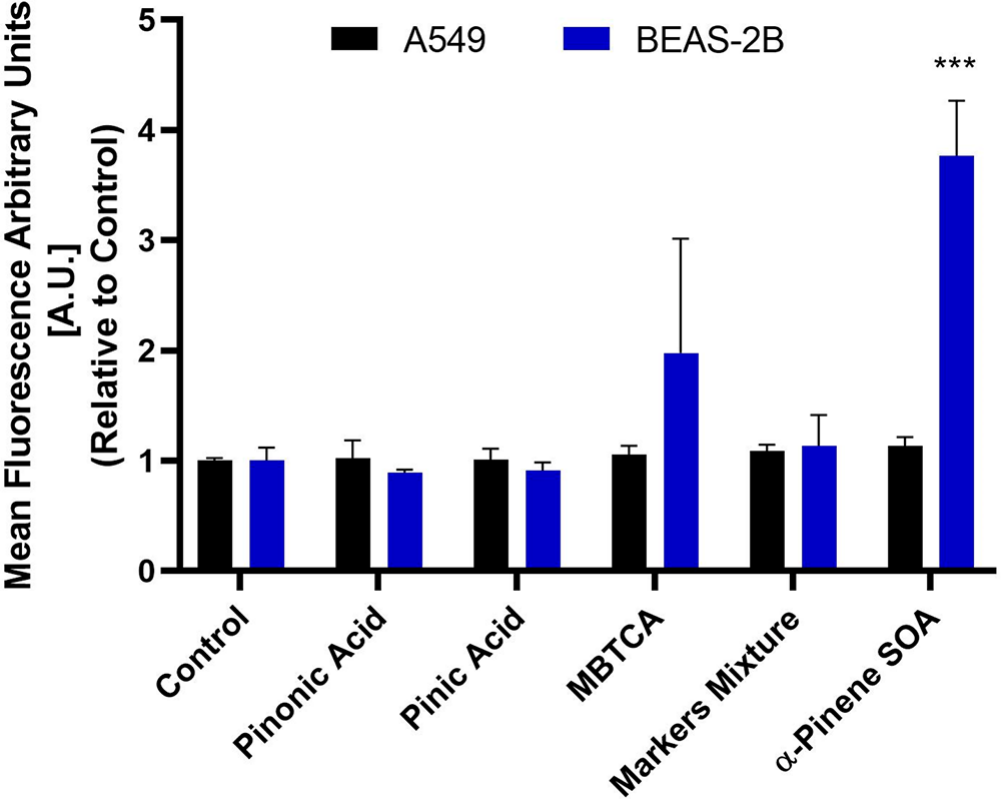
Fold change in general oxidative stress as a measure of carboxy-H_2_DCFDA reduction signal relative to control. Measurements were made in A549 and BEAS-2B cell lines 6 h postexposure with α-pinene SOA molecular tracers, their equimolar mixture, and the α-pinene ozonloysis SOA through the flow cytometer. The change in signal, as determined through three independent experiments, were normalized to controls and a one-way ANOVA followed by Dunnett’s post hoc test was performed on the data set to determine the statistical significance of change in signal when compared with untreated controls. The *p*-value <0.05 was considered statistically significant for our analysis where *** indicates a *p*-value ≤0.001.

The cell lines were further assessed for changes in general oxidative stress using flow cytometric analysis and stained with carboxy-H_2_DCFDA (i.e., ROS indicator) and propidium iodide (i.e., viability indicator). The percentage of live cell populations were observed to determine an overall change in oxidative stress within the cells that could be attributed to cell death. Since we observed cellular death in α-pinene ozonolysis SOA treated BEAS-2B cells after 24 h treatment (Figure 3), the 6-h time point was selected to determine whether the ROS buildup attributed to the cellular death, as previous studies demonstrated the peak time ROS build-up to be around 4–8 h.^56^ The gating strategy, applied through exclusion of cellular aggregates through FSA/FSH dot plot and further removal of PI+ cells from the final analysis, allowed us to include only single live cells in the analysis. As shown in Figure 5, A549 and BEAS-2B cells were treated with the highest aerosol exposure concentration of 200 μg mL^–1^, resulting in the graph shown as a change in the mean fluorescence signal relative to the untreated controls. The full SOA mixture generated from α-pinene ozonolysis induced almost a 4-fold increase in the ROS-associated signal for the BEAS-2B cells exposed, suggesting an imbalance of oxidative stress response in cells within a few hours after exposure. The statistical analysis revealed MBTCA-treated cells was not significant when compared with untreated baseline controls. The remaining single-component exposures of the α-pinene SOA molecular tracers and their equimolar mixtures did not contribute toward any significant ROS changes in both the A549 and BEAS-2B cells. This indicates the presence of certain SOA components, found in the α-pinene ozonolysis SOA, might be important contributors toward cellular death. As a result, we carefully examined the RPLC/ESI-HR-QTOFMS negative and positive ion mode data collected from the α-pinene ozonolysis SOA sample for other SOA constituents other than pinic acid, pinonic acid, and MBTCA.

### 3.5 RPLC/ESI-HR-QTOFMS Analysis of PAM-Generated α-Pinene SOA

**Figure 6:**
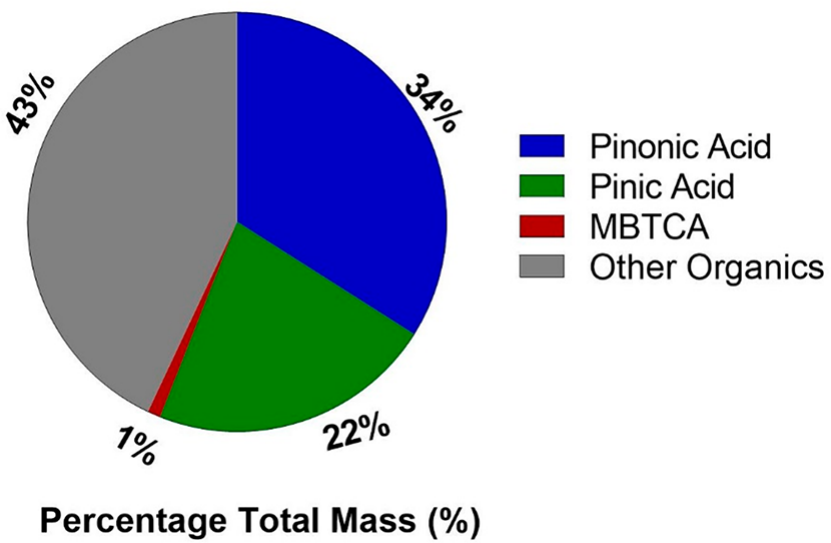
Pie chart showing the RPLC/ESI-HR-QTOFMS measurements of pinic acid, pinonic acid, and MBTCA accounted for ∼57% of the total SOA mass produced from α-pinene ozonolysis. SOA mass was determined by multiplying the total SOA volume measured in real-time by the scanning mobility particle sizer (SMPS) by the previously reported SOA density of 1.2 g cm^–3^.^63^

α-Pinene ozonolysis SOA showed increased ROS and cytotoxicity relative to pinic acid, pinonic acid, and MBTCA. As shown in Figure 6, these latter three α-pinene SOA molecular tracers account for ∼57% by mass of the total SOA mass collected. Docherty et al.^31^ and Surratt et al.^57^ reported that organic peroxides of unknown molecular composition generated from α-pinene ozonolysis account for ∼47 and 49%, respectively, of the total SOA mass. Here, we hypothesize that organic peroxides present in high yields^58−60^ within α-pinene ozonolysis SOA are responsible for increased ROS and cytotoxicity content based on qualitatively similar trends observed for isoprene-derived hydroperoxides.^61^ Organic peroxides (i.e., hydroxyhydroperoxides) present in our α-pinene ozonolysis SOA were identified using RPLC/ESI-HR-QTOFMS operated in the positive ion mode. As shown in Table 1 and Figure S7 (SI), seven multifunctional organic hydroperoxides are present in the SOA mass generated from α-pinene ozonolysis. These seven organic hydroperoxides potentially contributed toward increased cytotoxicity not observed with pinonic acid, pinic acid, and MBTCA.

**Table 1:**
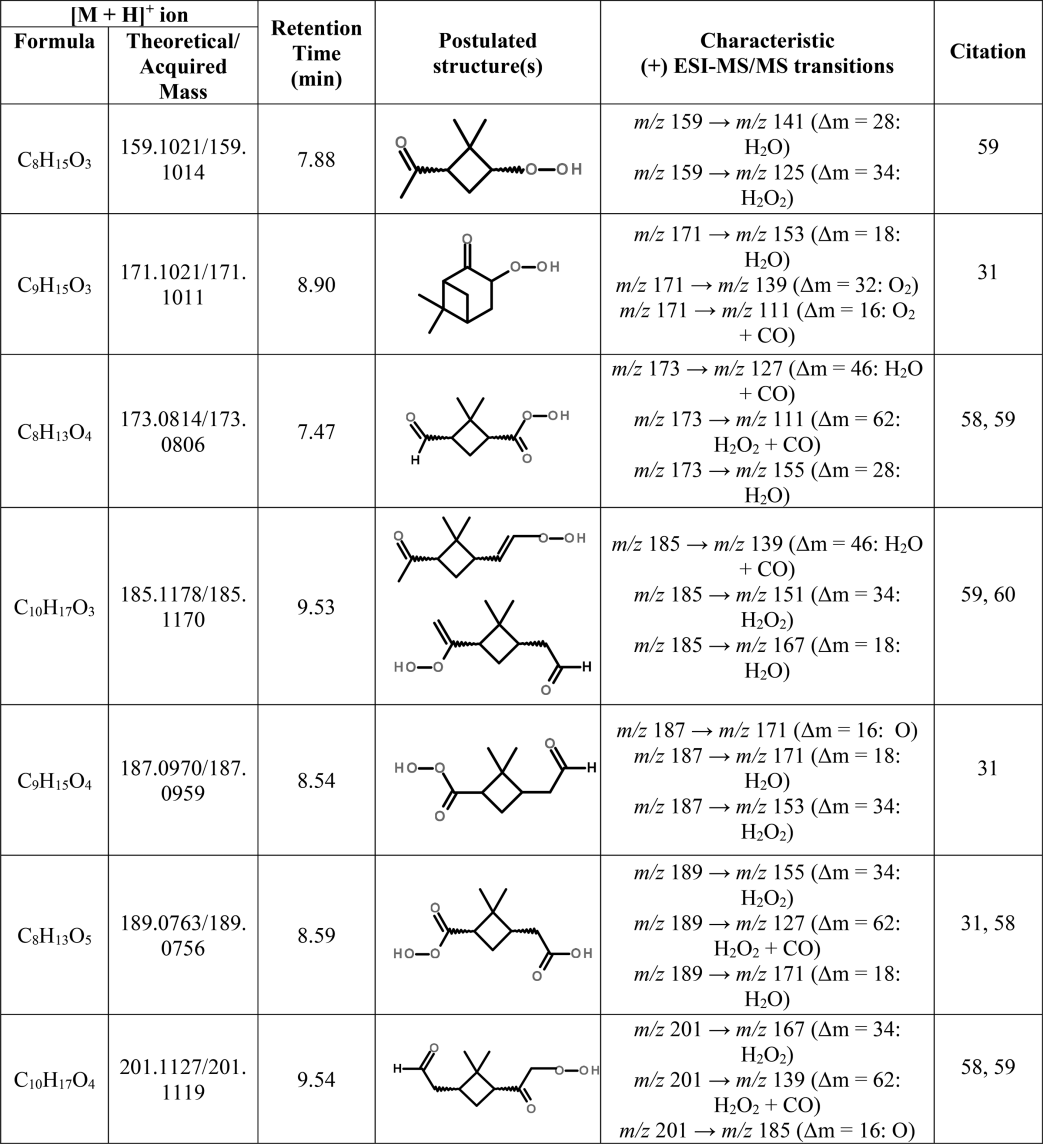
Organic Hydroperoxides Identified in the SOA Mass Obtained from the Ozonolysis of α-Pinene in the PAM Reactor

To further support the chemical characterization of the α-pinene-derived hydroperoxide structures shown in Table 1 and Figure S7, we analyzed a 1,2-ISOPOOH (2-hydroperoxy-2-methylbut-3en-1-ol) authentic standard (which has a molecular weight of 118 g mol^–1^) by the RPLC/ESI-HR-QTOFMS method operated in the positive ion mode as a surrogate for the proposed α-pinene-derived organic hydroperoxides. By analyzing this model hydroperoxide standard with RPLC/ESI-HR-QTOFMS, we were able to determine how the hydroperoxides ionize and their typical neutral losses produced during MS/MS experiments. As shown in Figure S8, we found that 1,2-ISOPOOH was not only retained and detected as the [M + NH_4_^+^]^+^ ion at mass-to-charge ratio (*m*/*z*) 136 by the RPLC/ESI-HR-QTOFMS positive ion mode method, but it also produced neutral losses of 35 (i.e., loss of H_2_O + NH_3_) and 51 (i.e., loss of H_2_O_2_ + NH_3_) during MS/MS experiments, which are consistent with the neutral losses we observed for the proposed structures shown in Table 1 and with a prior study by Zhou et al.^62^ that used atmospheric pressure chemical ionization-tandem mass spectrometry (APCI-MS/MS) operated in the positive mode.

As shown in Table 1, organic hydroperoxides were measured from this same SOA using RPLC/ESI-HR-QTOFMS operated in the positive ion mode (Figure S9), which likely explains some portion of the “other organics” not quantified at the molecular level by RPLC/ESI-HR-QTOFMS. Since we lack authentic standards for these organic hydroperoxides, we were not able to currently estimate their individual contributions to the SOA mass.

## 4 Discussion

This study reports a time-dependent response of pinic acid/pinonic acid/MBTCA and their equimolar mixture and α-pinene ozonolysis SOA under increasing exposure concentrations. The MTT assay results revealed that pinic acid, pinonic acid, MBTCA, and their equimolar mixtures did not exhibit significant cellular toxicity and inhibition between the 0.01–200 μg mL^–1^ dose range for up to 48 h of exposure in both A549 and BEAS-2B cells. A previous study by Gangwal et al.^64^ used a multiple path particle dosimetry (MPPD) model to estimate the lung uptake of ultrafine particles (diameters ≤100 nm) ranges from 0.006 to 0.02 μg cm^–2^ to a particle concentration of 100 mg m^–3^ at 24 h exposure time.^64,65^ To put these dose ranges in atmospheric context, we note that the lifetime uptake dosage of atmospheric ultrafine particles in the lungs is approximately 6.6 μg cm^–2^.^66^ At an average daily inhaled volume of air equal to 25 m^3^,^67^ alveolar lung surface area of 100 m^2^, an aerosol particle deposition efficiency of 30% (∼50% for ultrafine particles),^67^ and an average mass concentration of ultrafine particles of 7.0 to 65.8 μg·m^–3^,^68^ the corresponding average daily lung exposure is approximately 7.5 × 10^–4^ μg cm^–2^ in “hot spot” (forested and rural) regions and 7.5 × 10^–5^ μg cm^–2^ in other atmospheric conditions as reported in Paur et al.^66^ The 24- and 72-h clearance of inhaled ultrafine particles from the alveolar region is assumed to be negligible.^67,69^ The maximum exposure concentration used in the study is 200 μg mL^–1^ which corresponds to 62.5 μg cm^–2^ of total cell exposure. At 7.5 × 10^–4^ μg cm^–2^ day^–1^, this concentration is achieved in ∼228 years of equivalent atmospheric exposure. The exposure dosage in the studied cell lines was higher than the environmentally relevant condition for acute exposure, helping us to assess the safety-index, i.e., the minimum concentration at which the α-pinene SOA and its markers can be exposed without significant contribution to ROS in human lung cells. Our results suggest that freshly generated α-pinene ozonolysis SOA might not induce significant cytotoxicity upon acute exposure at environmentally relevant conditions; however, chronic exposure studies are important to preclude long-term inhalation effects of the α-pinene ozonolysis SOA system. In addition, the α-pinene SOA constituents we report here can also contribute to the aged particles^70^ which form as a result of chemical interaction between fresh α-pinene SOA and anthropogenic pollution.^1,2^ These may add to the increased ROS responses within lung cells during acute exposures and warrant further studies.^18,19^

Our study demonstrates that α-pinene ozonolysis SOA induced a clear cytotoxic response at the 200 μg mL^–1^ exposure concentration. Specifically, cellular viability in BEAS-2B cells decreased to 78% and 44% at 24 and 48 h, respectively, of exposure time. A study by Møller et al.^71^ reports that ultrafine particles of diameters ≤100 nm accumulate in the lung periphery, exhibiting 96% retention after 24 h of exposure and less clearance through the liver after 48 h of exposure. This suggests, even with lower exposure concentrations, that lifetime exposures might lead to similar cellular fates as exhibited by the 200 μg mL^–1^ exposure concentration. The increased PI staining from the α-pinene SOA shown in Figure 3 demonstrated that the decrease in proliferation (%) was due to cellular death and not cellular growth/metabolic inhibition.

While BEAS-2B cells exhibited cellular toxicity following exposure to α-pinene ozonolysis SOA, A549 cells did not. These results are consistent with previous acellular (or chemical-based assay) oxidative stress studies, where low dithiothreitol (DTT) activity in α-pinene SOA was measured when compared with other biogenic and anthropogenic SOA systems.^39^ No significant change in toxicity was observed with increasing mass of exposure of α-pinene ozonolysis SOA in A549 cells, implying in vitro cytotoxicity observed at higher concentration depends on the metabolic activity type of the cell lines.^72^ Increasing the exposure time from 24 to 48 h also did not induce significant changes in the toxicity profile in A549 cells. A notable part of this study was the MBTCA exposures in A549 cells. Cellular metabolic activity increased to 140% after exposure to 1 μg mL^–1^ of MBTCA and decreases with increasing exposure concentration and time. This suggests dosage-dependent metabolic stress induced by MBTCA in A549 cells,^72^ as shown in Figure 2c.

The differential response between cell lines could have been attributed to the inherent nature of A549 cancer cells that do not follow normal growth patterns of BEAS-2B (untreated) cells. Another reason could be the fact that two cell types respond differently to pro-inflammatory tumor growth factor (TGF-β) stimuli.^73^ MBTCA may also cause a slight increase in A549 cellular proliferation as the actively dividing cells support energy-dependent respiration processes that use tricarboxylic acids (TCA) as intermediate substrates. The evidence suggests that MBTCA could act as a surrogate for TCA.^74^ Furthermore, all cancer cells exhibit a hallmark of cancer known as the “Warburg effect” that is characteristically associated with higher glucose consumption, and higher rate of respiration.^75^ Thus, MBTCA provided the intermediate reactants to support a higher rate of respiration and increasing cellular proliferation rate. Since MTT is a pH sensitive compound, its darker color change is dependent on carbon dioxide production during enhanced respiration rate of A549 cell.^76^

The alveolar and bronchial cells seemed to be insensitive to the molecular tracers of α-pinene SOA formation. Previous exposure studies with environmental pollutants (formaldehyde) in both BEAS-2B and A549 cells revealed that the acute exposure, without prior sensitization, did not induce significant changes.^77^ But after sensitization with TNF-α, both cell lines responded differently to the environmental toxins (formaldehyde).^77^ Another study revealed that BEAS-2B cells tend to be more sensitive to PM_10_ and PM_2.5_ exposure at the same dosage as that of A549 cells.^78^ The difference in cell cycle and metabolic response could be attributed to overexpression of lung resistance-related protein (LRP) in A549 cells. At cytotoxic concentrations of drug/metabolite exposures, LRP levels remain unchanged.^79^ However, increased LRP stimulation is observed at lower dosage concentrations and increasing exposure time.^79^ A549 cells also have more antioxidant properties (Nrf2 expression), enhanced metabolic stress, and increased cellular proliferation following endogenous chemical exposure.^80^ The increase in cellular proliferation at lower concentrations could be attributed to A549 cells’ resistance to NRF-2 gene regulation. Homma et al.^80^ reported that A549 cells are more resistant to drug exposure due to a mutation in the KEAP-1-NRF-2 (Antioxidant response gene) system. This mutation makes the A549 cells more resistant to external compound exposure and NRF-2 activation increases the cellular proliferation rate. The dysregulation of NRF-2 response system in A549 cell lines renders it more resistant to SOA exposure than BEAS-2B cell lines. As previous work by Lin et al.^81^ suggests activation of NRF-2 related genes after exposure to isoprene-derived SOA in BEAS-2B cells, we speculated similar antioxidant response in cells following α-pinene SOA exposure. The effect is not evident in A549 cells due to dysregulation of NRF-2 response in these cell types. Furthermore, peroxiredoxin (Prx), an antioxidant protein that is known to overexpress in A549 cells, likely attributed to the differences in the cellular responses when compared to BEAS-2B cells.^82^ All these factors could contribute to the differences in metabolic activity observed in A549 cells when compared with BEAS-2B cells. LRP expression at the lower exposure concentration of MBTCA and increasing exposure time could be attributed toward higher metabolic activity at 1 and 0.01 μg mL^–1^ exposure dosages at 24 and 48 h, respectively. Another study revealed that the exposure of an inhalable plasticizer led to the enlarged morphology of A549, increased proliferation, cell progression, and the loss of epithelial structure in a dosage-dependent manner.^83^ This dosage-dependent exposure effect is lost with increased dosage and cells regain their morphology and metabolic rate at higher dosage. A549 cells become adaptive and refractory at higher concentrations used, hence limited changes in viability were observed at the 100–200 μg mL^–1^ concentration range of MBTCA. This concentration-resistance of A549 cells is a characteristic behavior, as shown in previous studies where cells become unresponsive to drugs after some time.^84^ Our results suggest MBTCA might induce metabolic changes in A549 cell lines at lower dosages, and time points shorter than 24 h are worth investigating in future studies.

Additionally, we investigated the oxidative stress response of BEAS-2B and A549 cells following exposure to pinonic acid/pinic acid/MBTCA, their equimolar mixtures, and α-pinene ozonolysis SOA. We did not observe any changes in ROS buildup within BEAS-2B or A549 cells when pinic acid, pinonic acid, and/or MBTCA were exposed to these cells. An approximate 4-fold increase in carboxy-H_2_DCFDA signal was observed in BEAS-2B cells treated with α-pinene ozonolysis SOA compared to the untreated control cells. This suggested that the cellular toxicity observed at 24 and 48 h after the treatment was due to increased buildup of ROS in 6 h post exposure. Other studies reveal that photochemical aging of SOA samples and loss of functionality of the SOA precursor might be attributed to ROS activity within the cells.^20,85^ Previous studies reported increased pro-inflammatory gene expression, i.e., interleukins 6 and 8 (IL-6, IL-8) and tumor necrosis factor-α (TNFα) when treated with α-pinene derived SOA.^20,22,23^ Similarly, both heme oxygenase-1 (HMOX-1) and interleukin-8 (IL-8) genes involved in antioxidative stress and anti-inflammatory responses through NRF-2/KEAP-1 pathway, were slightly altered in the previous study by Ito et al.,^21^ when BEAS-2B and U937 macrophages were treated with freshly aged α-pinene ozonolysis SOA (Table S1). However, an increase in ROS signals was not observed in A549 cells with similar treatments and concentrations, indicating that the oxidative stress response changes substantially with cell lines. Our current study compliments the study by Chowdhury et al.,^18^ where increases in ROS was not observed in the A549 cells following exposure for both aged and fresh SOA generated from α-pinene ozonolysis. However, our study highlights the use of noncancerous cell line models, such as BEAS-2B cells, to study the exposure effects, as A549 cells exhibited different cellular metabolism and dosage response than normal lung cells. Thus, careful selection of appropriate cell line models is important for toxicological assessments of SOA because the use of A549 cells by themselves may not provide sufficient information.^18,19^

Taken together, these observations suggest that compounds other than pinonic acid, pinic acid, and MBTCA that are present in α-pinene ozonolysis SOA induce cellular toxicity. On the basis of previous studies, including work from our group on complementary systems,^54,59^ the most likely candidate compounds are multifunctional organic peroxides that are generated from α-pinene ozonolysis. Many prior studies have measured a significant organic peroxide contribution to SOA generated from α-pinene ozonolysis,^31,57^ including multifunctional organic peroxides.^15^ As a result, we carefully examined our RPLC/ESI-HR-QTOFMS data and found seven organic hydroperoxides (see Table 1) were detected at the molecular level in the full SOA mixture generated from α-pinene ozonolysis. Notably, pinic acid, pinonic acid, and MBTCA contributed to ∼57% of the total SOA mass generated, and thus, the organic hydroperoxides measured here likely contribute to the mass closure, but this remains uncertain due to the lack of available authentic standards. Yet, there are likely other unidentified organics in the SOA sample that may also add to the ROS and cytotoxicity. Organic hydroperoxides generated from α-pinene ozonolysis are likely attributed to ROS removal by lung antioxidants, and hence, A549 cells with higher antioxidant proteins exhibited less toxic response at the same dosage than the BEAS-2B cells. In another study, a positive correlation between the total organic peroxide concentration and ROS yield within surrogate lung fluid systems suggests that organic hydroperoxides may play an important role in ROS buildup from biogenic SOA.^86^

The prior work by Surratt et al.^61^ examined the toxicity of varying chemical types of isoprene-derived SOA in BEAS-2B cells. Of various types of isoprene-derived SOA components, hydroxy-hydroperoxides, such as 1,2-ISOPOOH, was found to induce the strongest DTT and gene expression responses related to oxidative stress pathways. Overall, these studies suggested that multifunctional organic hydroperoxides are likely amongst the strongest ROS responses within lung cells.

Our results support this hypothesis and imply that identified particulate organic hydroperoxides from α-pinene ozonolysis may induce significant increases in ROS after exposure to the lung model systems. However, due to the lack of available authentic organic hydroperoxide standards from α-pinene ozonolysis, we cannot fully rule out that other unidentified organics in the α-pinene ozonolysis SOA sample that may also add to the ROS and cytotoxicity. For example, if the organic hydroperoxides identified in this study could be synthesized, then toxicological assessment of these compounds could be conducted as well as the determinations of their mass contributions to the total SOA mixture generated from α-pinene ozonolysis.

## Supporting Information Available

Additional experimental details: Figure S1, information on the inhibitory concentration-50 (IC_50_) of SOA produced from α-pinene ozonolysis; Figures S2 and S3, graphs for the cellular proliferation (in %) for BEAS-2B cells and A549 cells, respectively, treated with equimolar mixtures of two selected α-pinene SOA molecular tracers in increasing concentration for 24 and 48 h; Figures S4–S7, inverted-phase microscopy images of pionic acid, pinic acid, and MBTCA treated A549 cells, respectively, at 24 and 48 h of treatment; Figure S8, six-point calibration curves for MBTCA, *cis*-pinonic acid, and *cis*-pinic acid generated by a negative ion mode RPLC/ESI-HR-QTOFMS analysis; Figure S9, RPLC/ESI-HR-QTOFMS positive ion mode analysis of the 1,2-ISOPOOH standard, demonstrating how exactly organic hydroperoxides are detected and fragment in during ESI-HR-Q-TOFMS analyses; Figure S10, RPLC/ESI-HR-QTOFMS positive ion mode analysis of the PAM-generated SOA from α-pinene ozonolysis that revealed the structures of organic hydroperoxides present during the exposures (seven of these structures were tentatively identified through the accurate mass measurements and tandem mass spectral (MS/MS) data); and Table S1, all the previous toxicology studies on α-pinene SOA, which are compared with the results presented in the current study (PDF)
