# Supplementary material for: Toxicological Responses of α-Pinene-Derived Secondary Organic Aerosol and Its Molecular Tracers in Human Lung Cell Lines
Source: Chem Res Toxicol. 2021 Mar 3;34(3):817–32. doi: 10.1021/acs.chemrestox.0c00409 (PMC7967287; doi:10.1021/acs.chemrestox.0c00409)
Supplement: Supplementary file 1 — tx0c00409_si_001.pdf [file tx0c00409_si_001.pdf]

Supplementary Information

Toxicological Responses of α-Pinene-Derived Secondary Organic Aerosol and its Molecular Tracers in Human Lung Cell Lines

Faria Khan[†], Karina Kwapiszewska[†], Yue Zhang[‡,§], Yuzhi Chen[‡], Andrew T. Lambe[§], Agata Kołodziejczyk[†,∥], Nasir Jalal[⊥], Krzysztof Rudzinski[†], Alicia Martínez-Romero[£], Rebecca C. Fry[‡], Jason D. Surratt[‡,i*], Rafal Szmigielski[†**]

[†] Institute of Physical Chemistry, Polish Academy of Sciences, Kasprzaka 44/52, 01-224 Warsaw, Poland

[‡] Department of Environmental Sciences and Engineering, Gillings School of Global Public Health, University of North Carolina at Chapel Hill, Chapel Hill, North Carolina, United States 27599

[§] Aerodyne Research Inc, Billerica, Masachusetts, United States, 01821

[∥]TROPOS (Leibniz-Institut für Troposphärenforschung, Permoserstrasse 15, 04318 Leipzig, Germany)

[⊥] Department of Interdisciplinary Science, Nanjing University of Information Science & Technology, Nanjing, Jiangsu, China, 210044

[£] Cytomics Core Facility. Príncipe Felipe Research Center. Avda. Eduardo Primo Yúfera, 3, 46012, Valenica, Spain

[i] Department of Chemistry, University of North Carolina at Chapel Hill, Chapel Hill, North Carolina, United States 27599

Corresponding Authors:

RS: ** ralf@ichf.edu.pl JDS: * surratt@unc.edu

	24 Hour Treatment	48 Hour Treatment
IC50	912.0	230.7
logIC50	2.960	2.363

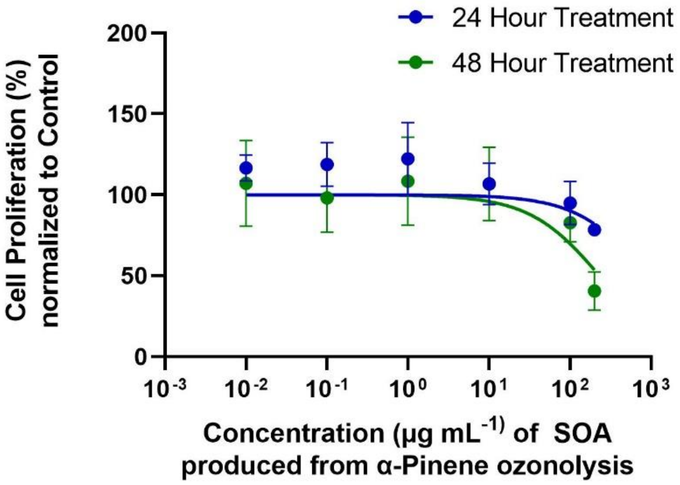

Figure S1. Inhibitory concentration-50 (IC_{50}) of SOA produced from a-pinene ozonolysis was found to be 912 and 230 µg mL^{-1} for 48 and 24 hours, respectively. $logIC_{50}$ was found to be 2.96 and 2.363 at the two different treatment time points in BEAS-2B cells. IC_{50} was calculated using GraphPad Prism (Version 8.00 for Windows, GraphPad Software, La Jolla California USA, www.graphpad.com).

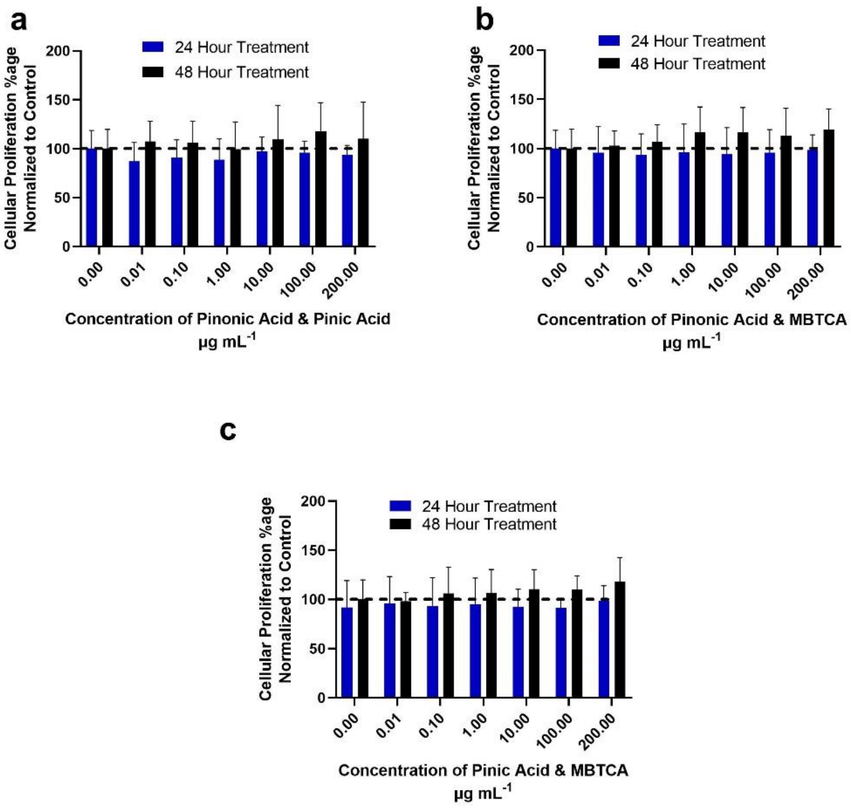

Figure S2. The cellular proliferation (in %) for BEAS-2B cells treated with equimolar mixtures of two selected α-pinene SOA molecular tracers in increasing concentration for 24 (blue bars) and 48 (black bars) hours: (a) pinonic and pinic acids; (b) pinonic acid and MBTCA; and (c) pinic acid and MBTCA.

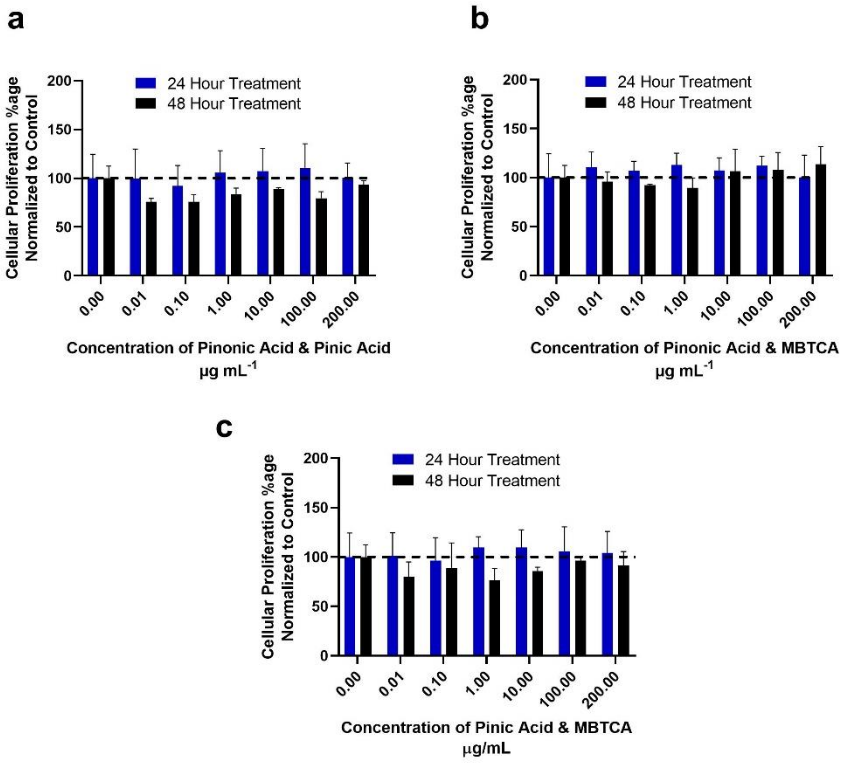

Figure S3. The cellular proliferation (in %) of A549 cell lines when treated with equimolar mixtures of two selected α-pinene SOA molecular tracers in increasing concentration for 24 (blue bars) and 48 (black bars) hours: (a) pinonic and pinic acids; (b) pinonic acid and MBTCA; and (c) pinic acid and MBTCA

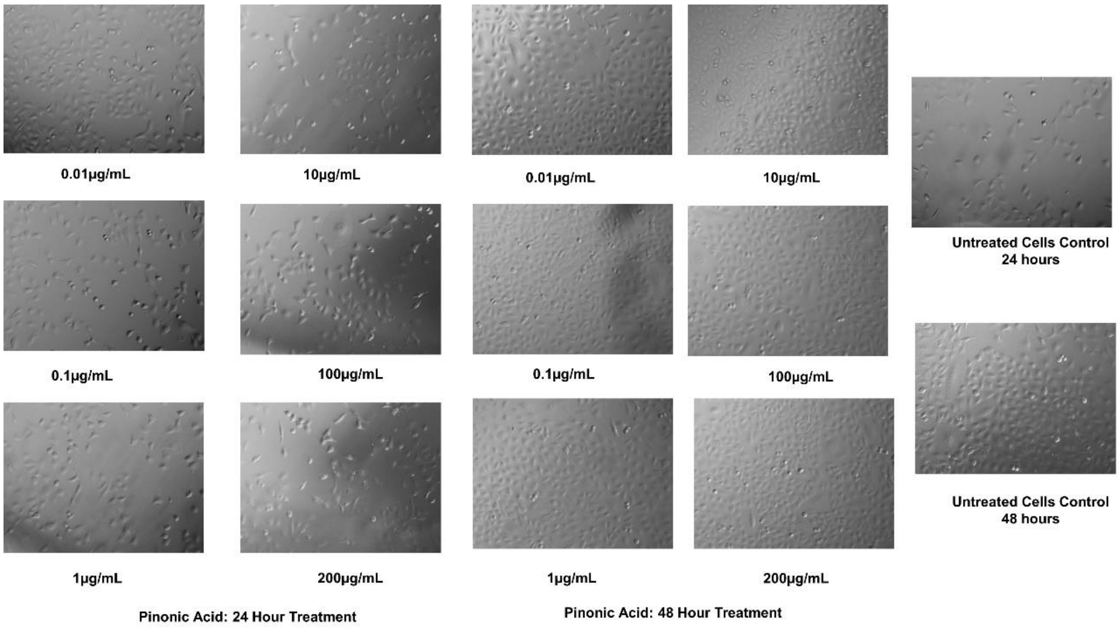

Figure S4. Inverted phase microscopy (Nikon Eclipse T1-SAM, Japan) images of A549 cells treated with increasing concentrations of pinonic acid at x100 magnification. The micrographs are scaled at 600 µm x 800 µm

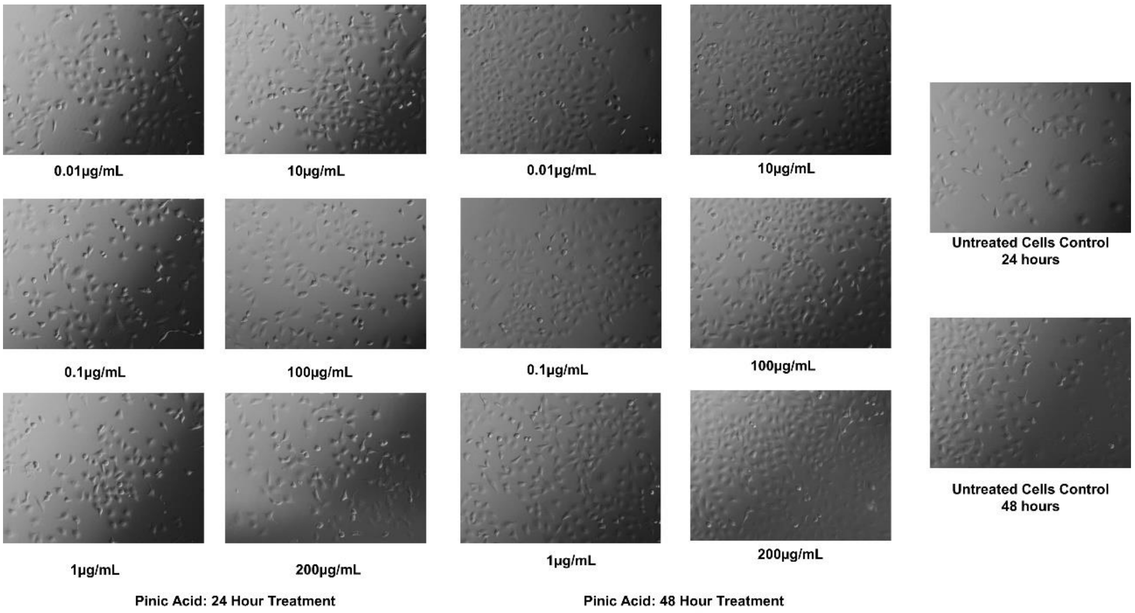

Figure S5. Inverted phase microscopy (Nikon Eclipse T1-SAM, Japan) images of A549 cells treated with increasing concentrations of pinic acid at x100 magnification. The micrographs are scaled at 600 µm x 800 µm

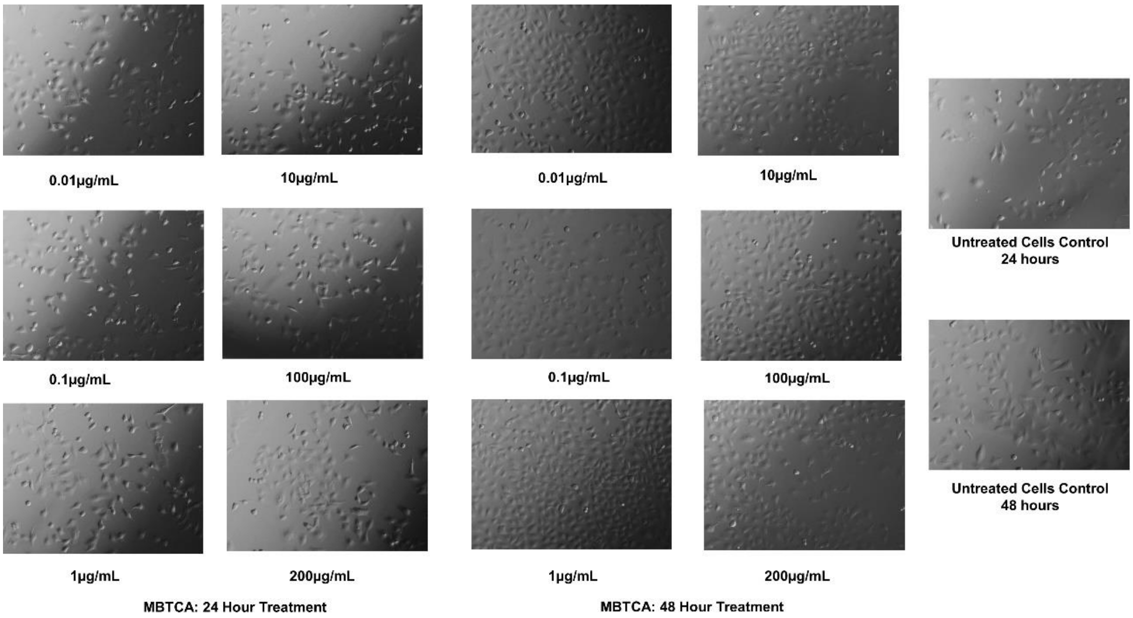

Figure S6. Inverted phase microscopy (Nikon Eclipse T1-SAM, Japan) images of A549 cells treated with increasing concentrations of MBTCA at x100 magnification. The micrographs are scaled at 600 µm x 800 µm

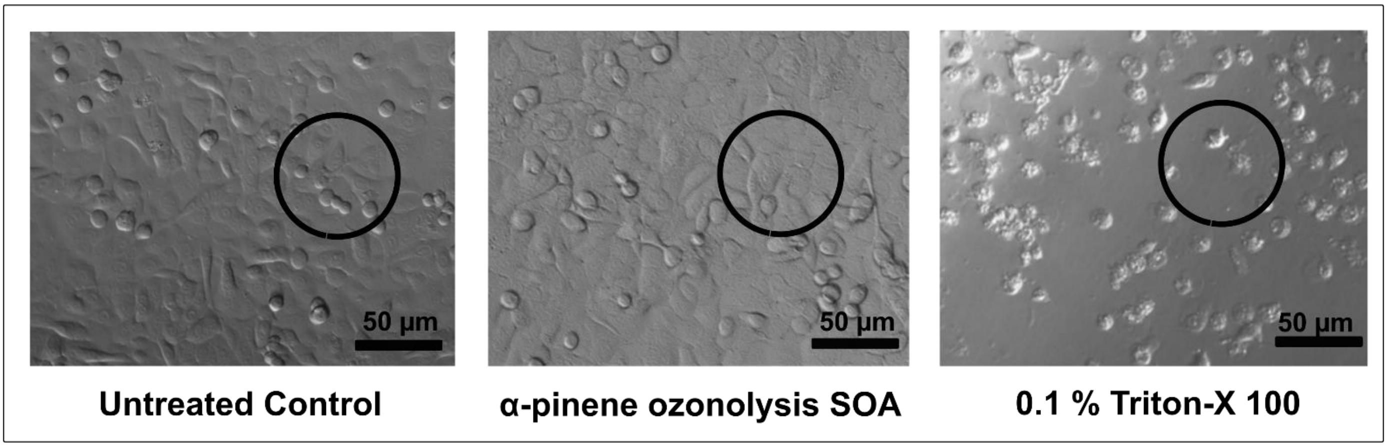

Figure S7: The phase contrast microscopy micrograph of A549 cells at 24 hours. The micrographs depict untreated control cells have similar morphology to α-pinene ozonolysis generated SOA treated A549. The positive control for cytotoxicity (Triton-X 100 treated cells) show visible signs of cellular degradation.

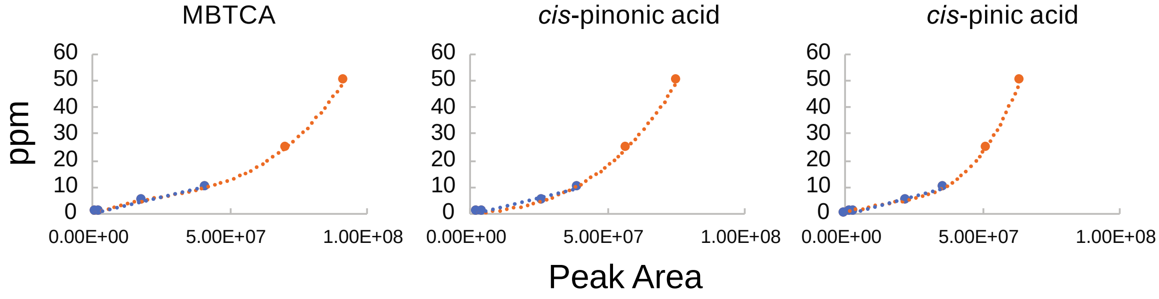

Figure S8. Six-point calibration curves for MBTCA, *cis*-pinonic acid and *cis*-pinic acid generated by RPLC/ESI-HR-QTOFMS negative ion mode analysis. Note that the data points within the linear range and the linear fit are shown in blue while the non-linear data points and the polynomial fit are shown in orange.

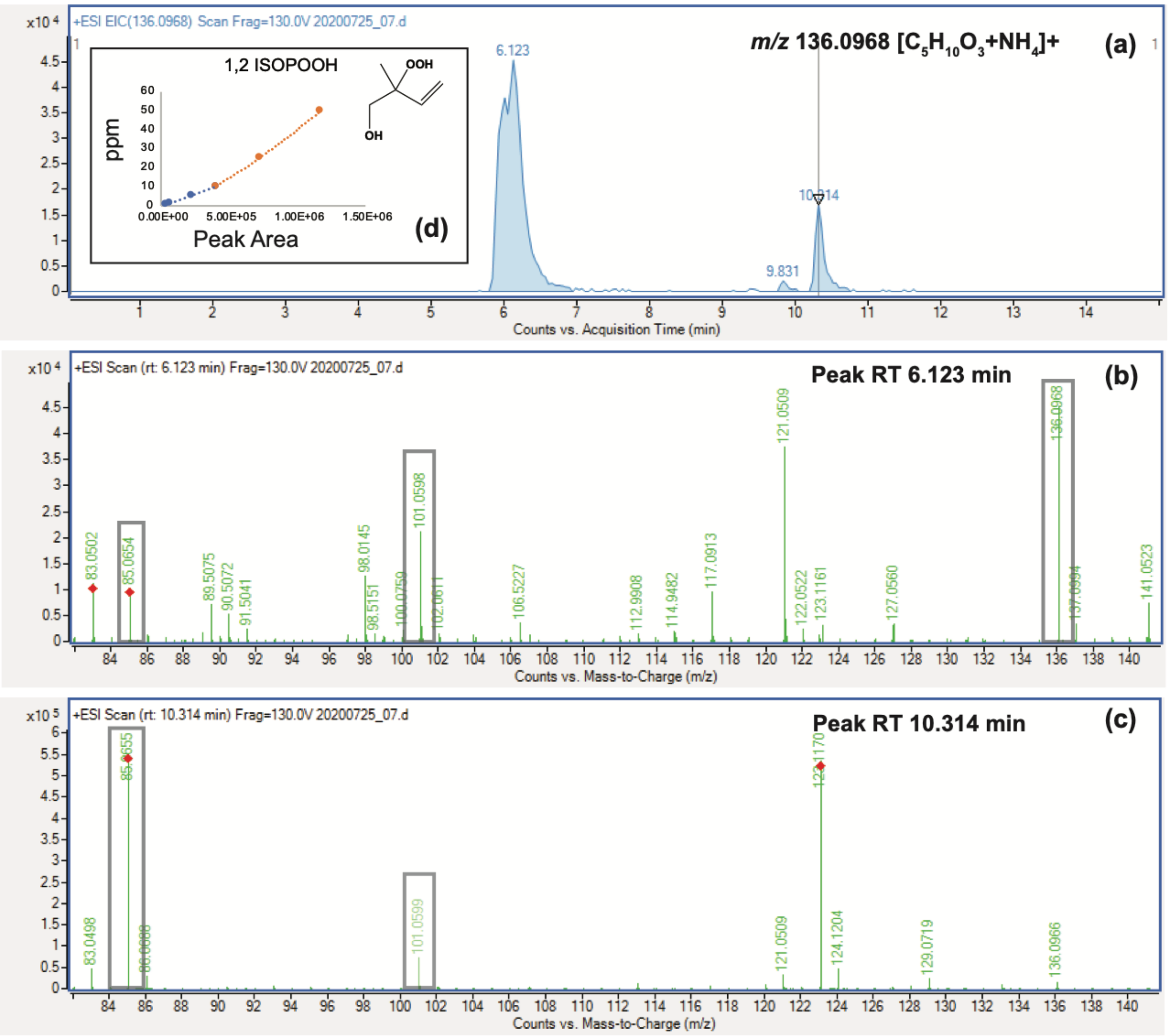

Figure S9. RPLC/ESI-HR-QTOFMS positive ion mode analysis of the 1,2-ISOPOOH standard demonstrates how organic hydroperoxides are detected and break down during our ESI-HR-Q-TOFMS analyses: (a) the extracted ion chromatogram (EIC) of $[M+NH_4]^+$ ion at mass-to-charge (*m/z*) 136 for 1,2-ISOPOOH; (b) the mass spectrum for chromatographic peak with the retention time (RT) of 6.123 min; (c) the mass spectrum for chromatographic peak with the RT of 10.314 min; (d) the six-point calibration curve for 1,2-ISOPOOH in the concentration range between 0.5 and 50 ppm. Consistent with our previous study, the $[M+H]^+$ molecular ion was not seen in the full MS scan given the -OOH group being a unfavorable protonation site within ESI. Instead, the dehydrated molecular ion $[M + H – H_2O]^+$ (*m/z* 101) was observed. The presence of the $[M+NH_4]^+$ ion resulted from background NH_4^+ contamination in our system. Therefore, the neutral loss of 35 u (i.e., NH_3 + H_2O loss from the $[M+NH_4]^+$ ion) may also contribute to the observed fragment ion at *m/z* 101. The fragment ion observed at *m/z* 85 can be explained by the neutral loss of 51 u (i.e., NH_3 + H_2O_2 loss of the $[M+NH_4]^+$ ion).

A)

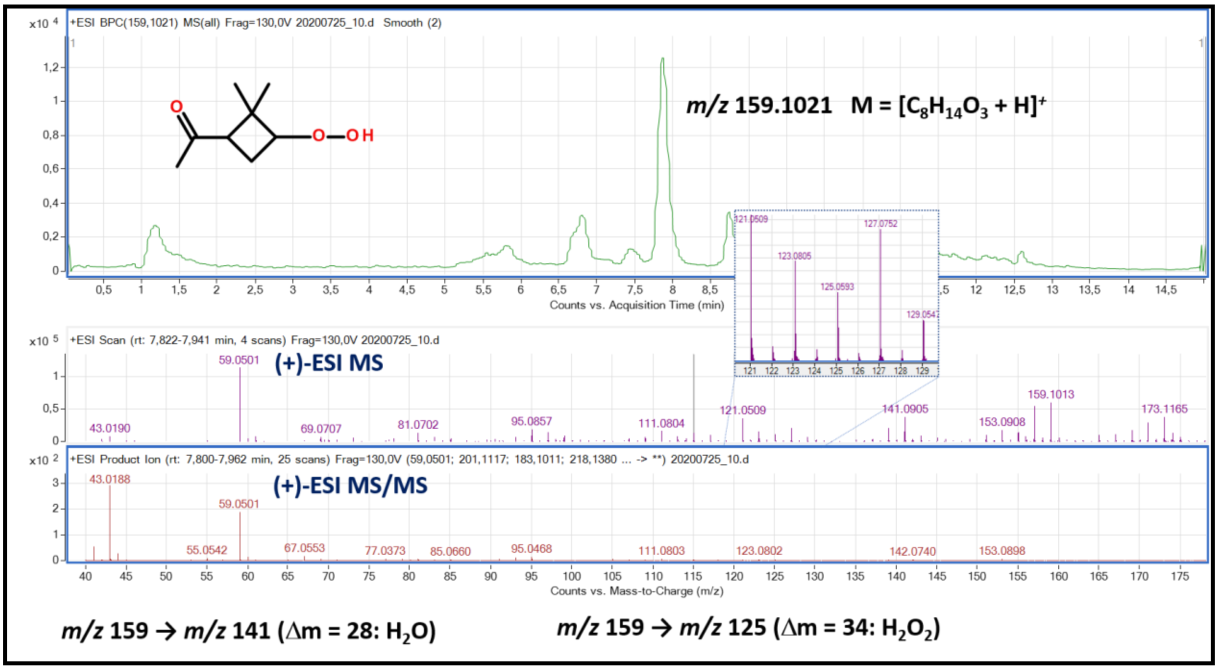
+ESI BPC(159,1021) MS(all) Frag=130,0V 20200725_10.d Smooth (2)
m/z 159.1021 M = [C8H14O3 + H]+
O—OH
Counts vs. Acquisition Time (min)
+ESI Scan (rt: 7,822-7,941 min, 4 scans) Frag=130,0V 20200725_10.d
(+)-ESI MS
59.0501
43.0190
69.0707
81.0702
95.0857
111.0804
121.0509
141.0905
153.0908
159.1013
173.1165
121.0509
123.0805
125.0593
127.0752
129.054
+ESI Product Ion (rt: 7,800-7,962 min, 25 scans) Frag=130,0V (59,0501; 201,1117; 183,1011; 218,1380 ... -> **) 20200725_10.d
(+)-ESI MS/MS
43.0188
59.0501
55.0542
67.0553
77.0373
85.0660
95.0468
111.0803
123.0802
142.0740
153.0898
Counts vs. Mass-to-Charge (m/z)
m/z 159 → m/z 141 (Δm = 28: H2O)
m/z 159 → m/z 125 (Δm = 34: H2O2)

B)

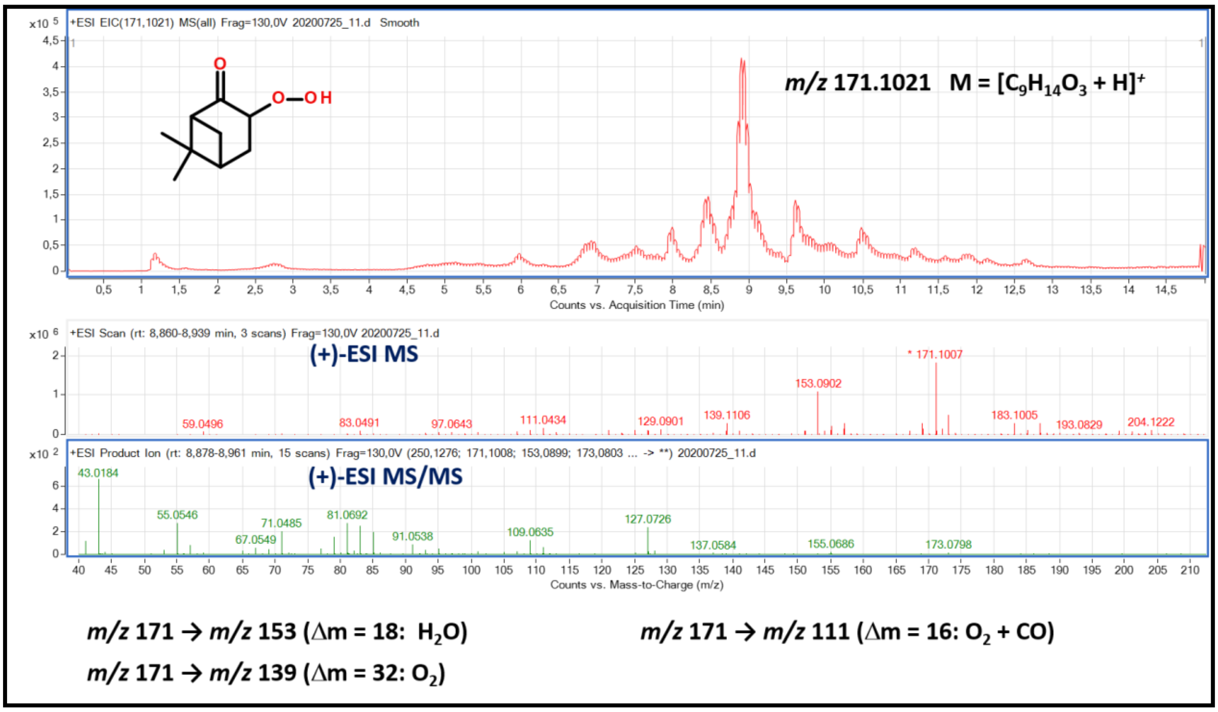
+ESI EIC(171,1021) MS(all) Frag=130,0V 20200725_11.d Smooth
m/z 171.1021 M = [C9H14O3 + H]+
O—OH
Counts vs. Acquisition Time (min)
+ESI Scan (rt: 8,860-8,939 min, 3 scans) Frag=130,0V 20200725_11.d
(+)-ESI MS
* 171.1007
153.0902
59.0496
83.0491
97.0643
111.0434
129.0901
139.1106
183.1005
193.0829
204.1222
+ESI Product Ion (rt: 8,878-8,961 min, 15 scans) Frag=130,0V (250,1276; 171,1008; 153,0899; 173,0803 ... -> **) 20200725_11.d
(+)-ESI MS/MS
43.0184
55.0546
71.0485
81.0692
67.0549
91.0538
109.0635
127.0726
137.0584
155.0686
173.0798
Counts vs. Mass-to-Charge (m/z)
m/z 171 → m/z 153 (Δm = 18: H2O)
m/z 171 → m/z 111 (Δm = 16: O2 + CO)
m/z 171 → m/z 139 (Δm = 32: O2)

C)

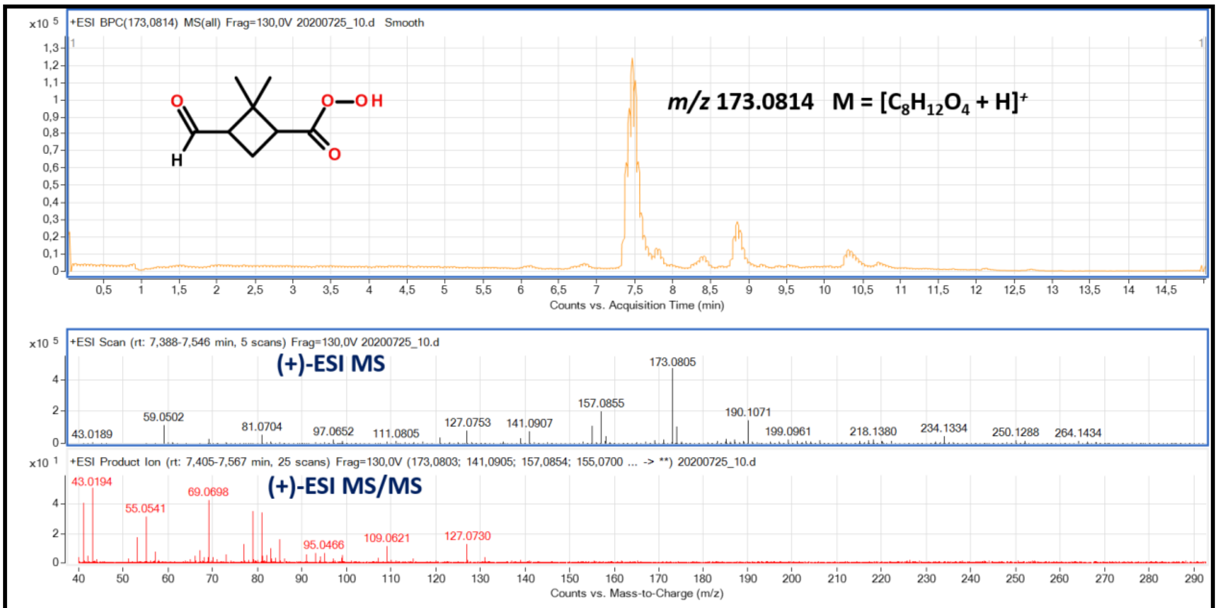

m/z 173 → *m/z* 127 (Δm = 46: H_2O + CO) *m/z* 173 → *m/z* 155 (Δm = 28: H_2O)

m/z 173 → *m/z* 111 (Δm = 62: H_2O_2 + CO)

D)

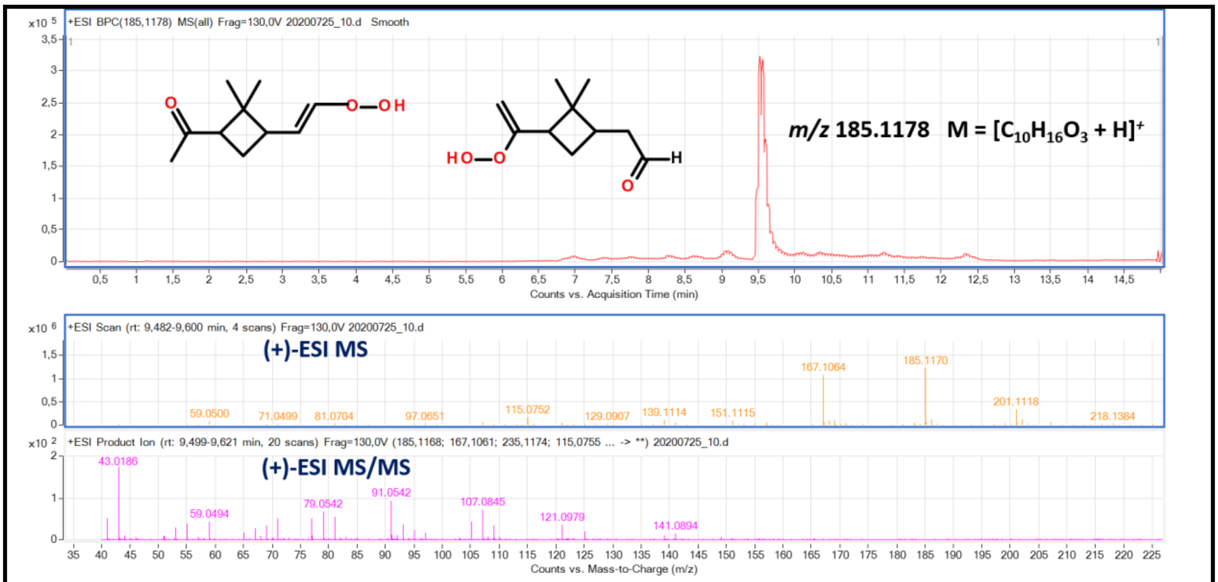

m/z 185 → *m/z* 139 (Δm = 46: H_2O + CO) *m/z* 185 → *m/z* 167 (Δm = 18: H_2O)

m/z 185 → *m/z* 151 (Δm = 34: H_2O_2)

E)

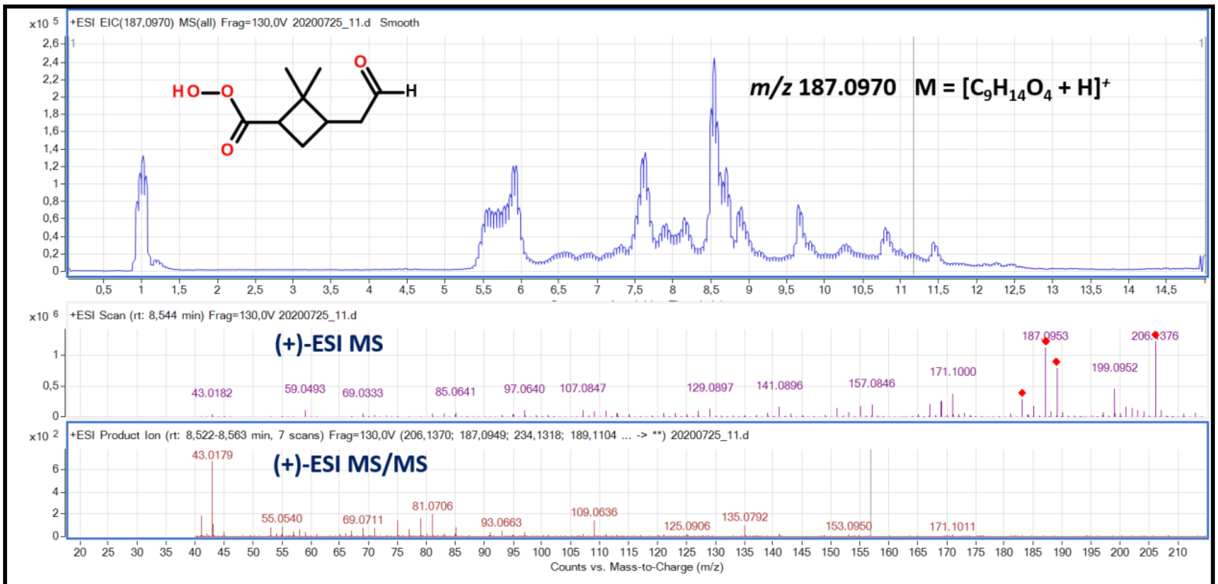

m/z 187 → *m/z* 171 (Δm = 16: O) *m/z* 187 → *m/z* 153 (Δm = 34: H_2O_2)

m/z 187 → *m/z* 171 (Δm = 18: H_2O)

F)

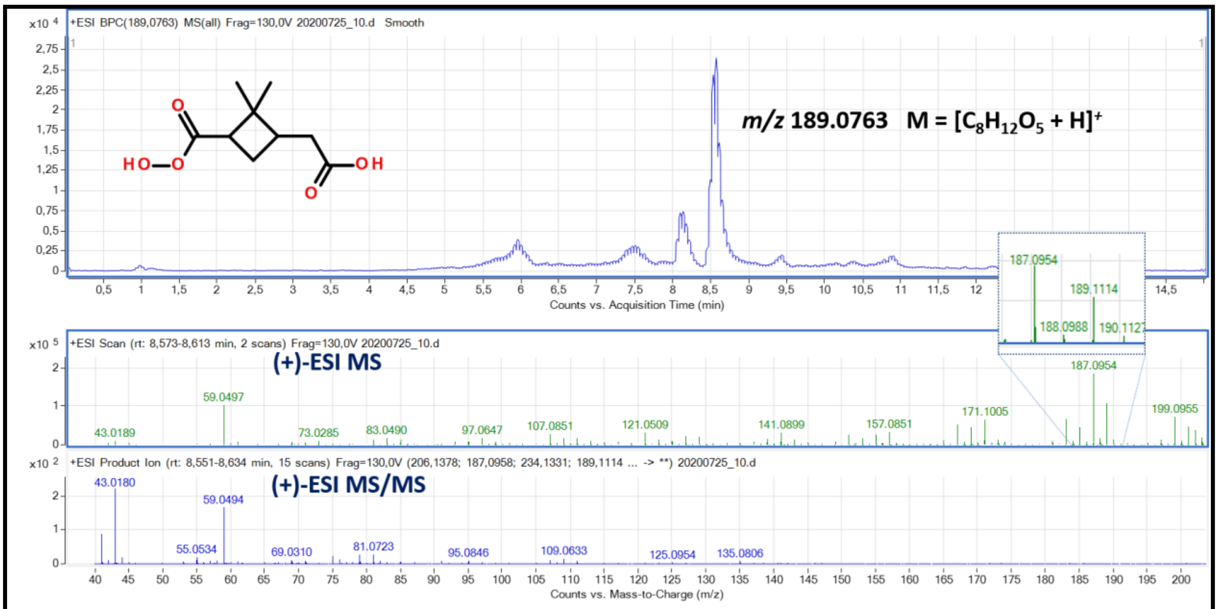

m/z 189 → *m/z* 155 (Δm = 34: H_2O_2) *m/z* 189 → *m/z* 171 (Δm = 18: H_2O)

m/z 189 → *m/z* 127 (Δm = 62: H_2O + CO + O)

G)

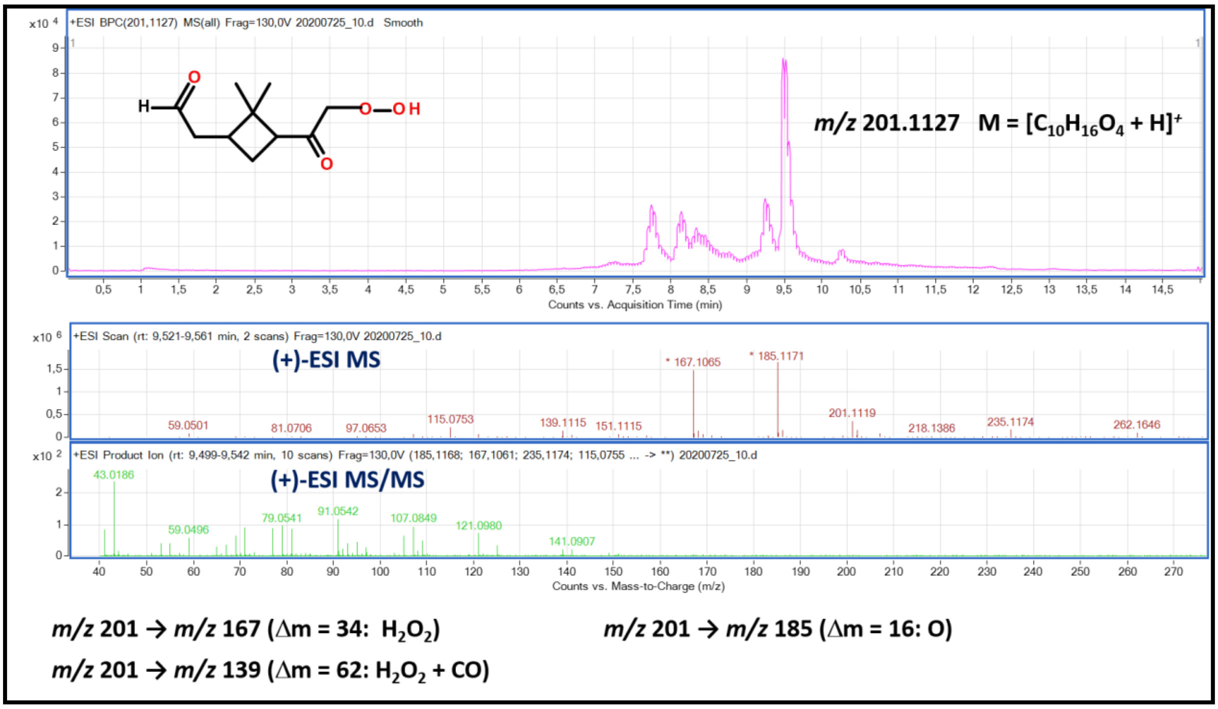

Figure S10. RPLC/ESI-HR-QTOFMS positive ion mode analysis data of the PAM-generated SOA from α-pinene ozonolysis revealed structures of organic hydroperoxides present during the exposures. Seven of these structures were tentatively identified through the accurate mass measurements and tandem mass spectra (MS/MS) spectra (figures A-G). Note that the first panel of each figure shows the extracted ion chromatogram for the $[M + H]^+$ ion associated with each organic hydroperoxide. The second panel is the positive electrospray mass spectrum for the $[M + H]^+$ ion and the third panel are the fragment ion mass spectrum (MS/MS) for the $[M + H]^+$ ion. .

Table S1. Comparison of previous toxicological studies associated with α-pinene SOA with the current study. Note that the fresh α-pinene SOA is generated through ozonolysis alone, while aged SOA is α-pinene SOA heterogeneously reacted with OH radicals.

α-Pinene SOA system studied	Model used	Response Type Studied	Toxicological End Point	Conclusions	Consistent with current Study	Reference
Pinonic Acid<br>Pinic Acid<br>MBTCA<br><br>Fresh α-pinene	A549<br>BEAS-2B	Cytotoxicity<br>Oxidative Stress<br><br>24 & 48 hours<br><br>0.01, 0.1, 1, 10, 100, 200ug/mL	MTT Assay<br><br>Calcein-AM/PI Staining<br><br>H_2DCFDA	α-pinene SOA at 200ug/mL induced high time-dependent cell death due to increased ROS	N/A	This Study
Fresh α-pinene	BEAS-2B	Lung Inflammatory response	IL-8 and Cytotoxicity	No significant change in IL-8<br>No toxicity	No	1
α-pinene+ NOx+ SOx	Macrophages	Lung Macrophage response	Cytotoxicty<br>IL-6, IL-8, and TNF-a<br>Phagocytic Activity<br>Wound Heal Assay	Decreased phagocytic activity	N/A	2
NOx+ NH_3 α -pinene;<br><br>SOx + NOx+ NH_3 α -pinene;	Apo E−/−) mice	Short term Cardiopulmonary response<br><br>7 days<br><br>250–300 mg/m3	gene expression of<br>TBARS<br>HO-1,<br>ET-1<br>MMP-9	SO_2: Increased expression HO-1, MMP-9, and ET-1<br>No SO_2: Decreased expression	N/A	3
NOx+ α -pinene;<br><br>SOx + NOx+ α -pinene;	Sprague-Dawley rats<br><br>ApoE−/− mice	Short term Cardiopulmonary response<br><br>200 μg m^{-3}<br>7 days	gene expression of TBARS<br>HO-1,<br>ET-1<br>MMP-9	Revealed limited biological response	N/A	4
NOx+ α-pinene<br><br>Fresh and Aged α-pinene	DTT acellular	Oxidative potential response	Oxidative stress	Negligible or no response	Yes	5
NOx+ aged α-pinene<br><br>Aged and Fresh α-pinene	Murine alveolar macrophages<br>0.1-10ug	Pro-inflammatory response and oxidative stress response	ROS/RNS production and levels of tumor necrosis factor-α (TNF-α) and interleukin-6 (IL-6)	Similar inflammatory response to all three conditions<br>Negligible or no response	Yes	6
Fresh & aged α-Pinene SOA	A549 cells<br>ALI exposure system<br>0-14μg	Cellular Viability	LDH Assay	More decreased viability in aged than fresh sample	Yes	7
Fresh, aged & NOx α-Pinene SOA	A549 cells<br>ALI exposure system	Cellular Viability<br>Oxidative stress<br>Superoxide	WST-1 assay<br>H_2DCFDA<br>ROS-Superoxide Detection Assay	No difference between NOx and NOx free system	Yes	8
Fresh α-Pinene SOA	BEAS-2B Cell Lines<br>U937 Cell Lines	Cellular Viability<br>Gene Expression (RT-PCR)<br>0.1, 1, or 10 μg/mL<br>3, 6 and 24 hours	GAPDH Ratio to:<br>HMOX<br>IL-8<br><br>ARE Activity	No significant change in viability<br>Slightly increased HMOX activity<br>IL-8 increase at 3 hours which is normalized at 24 hours<br>No change in ARE activity	Yes-consistent results at similar dosage	9

TBARS: thiobarbituric acid reactive substance, (HO)-1: heme-oxygenase, (ET)-1: endothelin, (MMP)-9: matrix metalloproteinase, (TNF-α): tumor necrosis factor-α, (IL-6): interleukin-6, (IL-8): interleukin-8 and antioxidant response element (ARE)

References

1. Jang, M.; Ghio, A. J.; Cao, G., Exposure of BEAS-2B Cells to Secondary Organic Aerosol Coated on Magnetic Nanoparticles. *Chemical Research in Toxicology* **2006,** *19* (8), 1044-1050.

2. Gaschen, A.; Lang, D.; Kalberer, M.; Savi, M.; Geiser, T.; Gazdhar, A.; Lehr, C.-M.; Bur, M.; Dommen, J.; Baltensperger, U.; Geiser, M., Cellular Responses after Exposure of Lung Cell Cultures to Secondary Organic Aerosol Particles. *Environmental Science & Technology* **2010,** *44* (4), 1424-1430.

3. Lund, A. K.; Doyle-Eisele, M.; Lin, Y.-H.; Arashiro, M.; Surratt, J. D.; Holmes, T.; Schilling, K. A.; Seinfeld, J. H.; Rohr, A. C.; Knipping, E. M., The effects of α-pinene versus toluene-derived secondary organic aerosol exposure on the expression of markers associated with vascular disease. *Inhalation toxicology* **2013,** *25* (6), 309-324.

4. McDonald, J. D.; Doyle-Eisele, M.; Campen, M. J.; Seagrave, J.; Holmes, T.; Lund, A.; Surratt, J. D.; Seinfeld, J. H.; Rohr, A. C.; Knipping, E. M., Cardiopulmonary response to inhalation of biogenic secondary organic aerosol. *Inhalation toxicology* **2010,** *22* (3), 253-265.

5. Tuet, W. Y.; Chen, Y.; Xu, L.; Fok, S.; Gao, D.; Weber, R. J.; Ng, N. L., Chemical oxidative potential of secondary organic aerosol (SOA) generated from the photooxidation of biogenic and anthropogenic volatile organic compounds. *Atmospheric Chemistry & Physics* **2017,** *17*, 839.

6. Tuet, W. Y.; Chen, Y.; Fok, S.; Champion, J. A.; Ng, N. L., Inflammatory responses to secondary organic aerosols (SOA) generated from biogenic and anthropogenic precursors. *Atmospheric Chemistry and Physics* **2017,** *17* (18), 11423.

7. Chowdhury, P. H.; He, Q.; Carmieli, R.; Li, C.; Rudich, Y.; Pardo, M., Connecting the Oxidative Potential of Secondary Organic Aerosols with Reactive Oxygen Species in Exposed Lung Cells. *Environmental Science & Technology* **2019,** *53* (23), 13949-13958.

8. Chowdhury, P. H.; He, Q.; Lasitza Male, T.; Brune, W. H.; Rudich, Y.; Pardo, M., Exposure of Lung Epithelial Cells to Photochemically Aged Secondary Organic Aerosol Shows Increased Toxic Effects. *Environmental Science & Technology Letters* **2018,** *5* (7), 424-430.

9. Ito, T.; Bekki, K.; Fujitani, Y.; Hirano, S., The toxicological analysis of secondary organic aerosol in human lung epithelial cells and macrophages. *Environmental Science and Pollution Research* **2019,** *26* (22), 22747-22755.
